# SorCS3 promotes the internalization of p75^NTR^ to inhibit GBM progression

**DOI:** 10.1038/s41419-022-04753-5

**Published:** 2022-04-07

**Authors:** Yanqiu Zhang, Yue Li, Yuhua Fan, Xiaoyuan Zhang, Zhihong Tang, Jing Qi, Baoshan Zhao, Fuyuan Li, Xiaofeng Chen, Huan Liang, Haiyan Xu, Dongliang Li

**Affiliations:** 1 Department of Basic Medical College, Harbin Medical University (Daqing), Daqing, China; 2 From the College of Medical Laboratory Science and Technology, Harbin Medical University (Daqing), Daqing, China; 3 State Key Laboratory of Cardiovascular Disease, Fuwai Hospital, National Center for Cardiovascular Diseases, Chinese Academy of Medical Sciences and Peking Union Medical College, Beijing, China; 4 Center for Endemic Disease Control, Chinese Center for Disease Control and Prevention, Harbin Medical University, Harbin, China; 5 Department of Neurosurgery, The First Affiliated Hospital of Harbin Medical University, Harbin, China; 6 Department of Clinical laboratory, Harbin Medical University Cancer Hospital, Harbin, China

**Keywords:** Tumour-suppressor proteins, Protein transport

## Abstract

Glioblastoma (GBM) is a fatal malignancy caused by dysregulation of cellular signal transduction. Internalization plays a key role in maintaining signalling balance. Previous reports showed that Sortilin related VPS10 domain containing receptor 3 (SorCS3) has the ability to regulate internalization. However, the impacts of SorCS3 on the biological processes involved in GBM have not yet been reported. In this study, we investigated the bio-function of SorCS3 in GBM. We found that SorCS3 was significantly downregulated in GBM. In addition, low expression level of SorCS3 predicted poor prognoses in patients with GBM. Here, we proved that SorCS3 suppressed cell invasion and proliferation mainly via NGF/p75^NTR^ pathway in GBM. We found that SorCS3 co-localized with p75^NTR^ in GBM cells and regulated the p75^NTR^ protein level by promoting trafficking of the endosomal to the lysosome. Immunofluorescence (IF) and Co-Immunoprecipitation (Co-IP) detection confirmed that SorCS3 bound to p75^NTR^, which subsequently increased the internalization of p75^NTR^, and then transported p75^NTR^ to the lysosome for degradation, ultimately contributing to inhibit of glioma progression. Taken together, our work suggests that SorCS3 is a marker of promising prognosis in GBM patients and suggests that SorCS3 regulates internalization, which plays a pivotal role in inhibiting glioma progression.

## Introduction

Glioblastoma Multiforme (GBM) is the most common type of primary intracranial tumour, with high mortality and recurrence rates and a low cure rate; it has a 5 year relative survival rate of only 5–8% [1]. Due to the specific location and high metastasis rate of glioma and the complexity of its molecular mechanism of occurrence and development, the prevention and treatment of GBM remains a global challenge, although it has been studied for decades. Hence, the study of GBM pathogenesis and discovery of key glioma biomarkers could facilitate the diagnosis and treatment of GBM recurrence.

Sortilin related VPS10 domain-containing receptor 3 (SorCS3) belongs to the vacuolar protein sorting 10 protein (VPS10p) receptor family. VPS10p domain receptors are a unique class of sorting receptors that direct the intracellular transport of target proteins between the cell surface, endosomes, Golgi and lysosomes in mammalian cells, and these proteins have receptor internalization abilities [2]. One of the characteristic functions of VPS10p family members is the regulation of ligand-induced internalization of receptors [3]. Analysis of the SorCS3 domain revealed that the short intracellular C-terminus contains consensus signals for rapid internalization [4]. We speculated that SorCS3 may have an internalization function similar to that of other VPS10p family members. In addition, receptor internalization plays an important role in regulating receptor signal transduction; thus, affecting internalization has the potential to influence cancer cellular function [5]. Earlier studies focused on roles of SorCS3 in the control of protein transport in neurons and disorders of systemic metabolism [6]. SorCS3 is a specific receptor for nerve growth factor (NGF) that regulates NGF signal transduction across membranes, and SorCS3 is mainly expressed in the hippocampus and cortex of the brain. However, the clinical relevance and physiological significance of the interaction between SorCS3 and NGF have not yet been fully elucidated.

Abnormal activation of signal transduction pathways, aberrant regulation of cell proliferation, and ectopic metastasis are key events in GBM development. Generally, dysregulated expression of growth factors and their receptors is the core component underlying abnormal activation of signal transduction pathways. NGF and NGF receptors are important in the progression of some neurological diseases, including tumours [7–9]. NGF regulates cell behaviour by binding to two different receptors, TrkA and p75^NTR^. In addition, it has been confirmed that p75^NTR^ is an important and potent mediator of invasion in human glioma. However, it is currently unclear whether the p75^NTR^ signal is propagated via internalization regulation or via other mechanisms.

In the present study, we revealed a novel molecular mechanism by which SorCS3 promotes internalization to regulate glioma progression. Our results revealed that SorCS3 interacts with p75^NTR^ but not TrkA. SorCS3 regulates p75^NTR^ by controlling its internalization from the plasma membrane to the lysosome, thereby limiting signal transduction, an essential driving force of tumour aggressiveness. Moreover, we found that low expression of SorCS3 is associated with a high degree of GBM malignancy. Hence, SorCS3 expression is a marker of favourable prognosis in GBM patients. Taken together, our results reveal a novel mechanism of regulated receptor signal transduction and suggest a promising receptor signal blocker for antitumour therapy.

## Materials and methods

### Cell lines and cell culture

The glioblastoma cell lines U-87 MG (U87) and U251 were purchased from the Type Culture Collection of the Chinese Academy of Sciences, Shanghai, China. The cell lines A172 and T98 were obtained from the First Affiliated Hospital of Harbin Medical University. Patient-derived cell lines were established from GBM resections, GBM tumour specimens were obtained from the Department of Neurosurgery, The First Affiliated Hospital of Harbin Medical University after obtaining informed consent, following guidelines, and permission from the Institutional Review Board. These cells were grown in DMEM (HyClone, SH3002201) supplemented with 10% FBS (Biological Industries, 04-001-1A) and 1% (vol/vol) penicillin/streptomycin (Beyotime, C0222) in an incubator (37 °C, 5% CO_2_). Patient-derived cell lines were established from primary resection specimen of GBM tumours (Fig. S2 Table 1). Briefly, tumour tissue was minced (by crossed scalpels) in DMEM cell culture medium supplemented with 10 % FCS, EGF, and FGF2, and passed through a cell strainer (Biosharp, BS70CS) to obtain a single-cell suspension.

**Table l:** Patient data of the tumours.

Age(years)	Gender	WHO Grade	Tumour type
47	Female	IV	Recurrent GBM

### TCGA and CGGA database analysis

By analysing publicly available datasets from The Cancer Genome Atlas (TCGA) and the Chinese Glioma Genome Atlas (CGGA), we examined the levels of SorCS3 and p75^NTR^ expression in human glioma samples. SorCS3 and p75^NTR^ levels were significantly changed in glioma samples compared with control samples.

### Colony formation assay

Cells (2000 cells/well) were plated in 6-well plates. After 7 days, colonies were counted under a microscope, fixed with 75% ethanol and stained with crystal violet staining solution.

### Transwell migration and invasion assays

Matrigel was diluted in serum-free medium at a volume ratio of 1:8. Twenty-four hours after transfection, cells (2 × 10^4^ cells) were seeded in the upper chamber. Then, 500 μl of complete medium was added to the lower chamber, and both chambers were incubated for 24 h. The upper chamber was transferred to 75% alcohol for fixation for more than 30 min and stained in crystal violet solution (0.5%) for more than 30 min.

### Western blot analysis

Total protein was extracted using RIPA buffer, and protein expression was analysed by western blotting as described previously [10]. Information about the antibodies utilized for western blotting is listed in Additional file 5: Table 2.

**Table 2:** Information of antibodies used in this study.

Antibody Name	Company	Application	Dilution	Host
**SorCS3**	R&D bio-techne	WB	1:1000	Goat
	Antibodies-Online	WB	1:500	Rabbit
	Bioss	WB	1:1000	Rabbit
**p75NTR**	Cell Signaling Technolog (CST)	WB	1:1000	Mouse
		Co-IP	1:50	Rabbit
		IF	1:600	Rabbit
**Akt**	CST	WB	1:1000	Rabbit
**p-Akt** (Ser473)	CST	WB	1:2000	Rabbit
**ERK**	CST	WB	1:1000	Rabbit
**p-ERK** (Thr202/Tyr204)	CST	WB	1:1000	Rabbit
**PCNA**	CST	WB	1:1000	Rabbit
**Snail**	ABcolonal	WB	1:1000	Rabbit
**E-cadherin**	CST	WB	1:1000	Rabbit
**Vimentin**	Wanleibio	WB	1:1000	Rabbit
**TrkA**	CST	WB	1:1000	Rabbit
**β-actin**	ProteinTech	WB	1:1000	Rabbit
**Flag**	ProteinTech	WB	1:1000	Rabbit
	Abcam	Co-IP	1:100	Rabbit
	CST	IF	1:100	Mouse
	ProteinTech	IF	1:100	Rabbit
**Rab7**	ProteinTech	WB	1:1000	Rabbit
**Rab5**	CST	IF	1:100	Rabbit
**EEA1**	ProteinTech	IF	1:100	Mouse
**LAMP2**	ProteinTech	WB	1:500	Rabbit
		IF	1:100	Mouse
**LAMP1**	CST	IF	1:200	Mouse
**GFAP**	ABcolonal	IF	1:100	Rabbit

### 5-FAM, SE-BSA internalization assay

5-FAM, SE (Invitrogen, C2210) is a fluorescent labelling reagent that exists as a single isomer to bind peptides, proteins and nucleotides via chemical reactions. According to the manufacturer’s protocol, fluorescently labelled bovine serum albumin (BSA, Sigma,A1933) was prepared by incubation in the dark. 5-FAM, SE-BSA collected from the cut-off column was filtered through a PVDF filter to remove bacteria. 5-FAM, SE-BSA was added to cultured glioma cells to observe the internalization ability under a fluorescence microscope.

### Co-immunoprecipitation

Co-IP was performed using a Pierce Co-IP Kit (Thermo Scientific) according to the manufacturer’s protocol. According to experimental needs, a purified anti-Flag antibody or anti-p75^NTR^ antibody was used to IP the antigen, and any co-immunoprecipitated interacting proteins were immobilized directly onto an amine-reactive resin by covalent coupling. The final protein eluate was denatured and analysed by western blotting.

### Nude mice tumorigenicity assay

Our study was approved by the Ethics Committee of Harbin Medical University (Daqing). Nude mice were randomly grouped into the control group and the OE-SorCS3 group. Mice were inoculated subcutaneously in the right flanks with 1 × 10^7^ U87 cells transfected with the negative control vector or the pCMV3-SorCS3-Flag plasmid.

### Statistical analysis

All values are expressed as the means ± SD, and all experiments were repeated at least three times. Student’s *t*-test was used to determine the statistical significance of the differences between groups. A comparative *t*-test was used for analysis of clinical samples. Statistical comparisons among multiple groups were carried out using analysis of variance (ANOVA) followed by Dunnett’s test. Differences with *P* < 0.05 were considered significant (*p < 0.05, **p < 0.01, ***p < 0.001).

## Results

### Low levels of SorCS3 expression in human GBM are associated with poor clinical outcomes

**Fig. 1:**
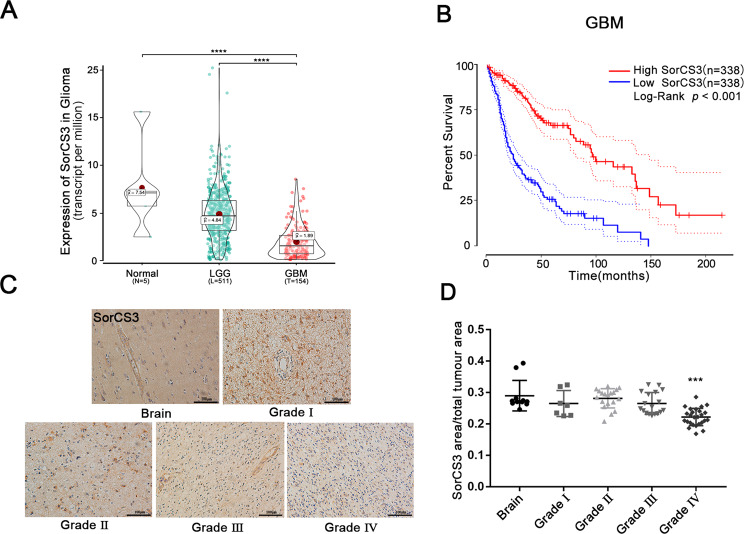
Overview of SorCS3 expression in TCGA glioma database. **A** SorCS3 expression level in adjacent normal tissues (*n* = 5), low grade glioma tissues (*n* = 511) and GBM (*n* = 154) in the glioma datasets from TCGA. **B** Kaplan-Meier Survival curves of different SorCS3 expression levels for GBM in TCGA dataset (High SorCS3, *n* = 338; Low SorCS3, *n* = 338; *p* < 0.001). **C**, **D** SorCS3 expression with different WHO grade glioma and normal brain tissues taken from 108 patients was assessed by IHC. Scale bar: 100 µm. *p < 0.05; **p < 0.01; ***p < 0.001, ns: not statistically significant.

To explore the potential roles of SorCS3 in glioma, we analysed RNA sequencing results from TCGA database and found that the mRNA level of SorCS3 was decreased in glioma, and we demonstrated that a low level of SorCS3 transcripts in low-grade glioma and high-grade glioma tissues was associated with shorter overall survival times in patients (Fig. 1A, B). Next, we performed immunohistochemical analysis on glioma tissue microarrays containing samples from 108 patients. We analysed SorCS3 expression according to grade, with grades I–IV corresponding to well-differentiated to poorly differentiated tumours. Low levels of SorCS3 protein were found to positively correlate with poorly differentiated in the tissue microarray analysis (Fig. 1C, D). In contrast to low expression, high expression of SorCS3 was significantly correlated with better survival (Fig. 1B). Taken together, these data demonstrated that SorCS3 is significantly downregulated in GBM, suggesting that it may function as a tumour suppressor.

### SorCS3 inhibits GBM cell proliferation, migration and invasion

**Fig. 2:**
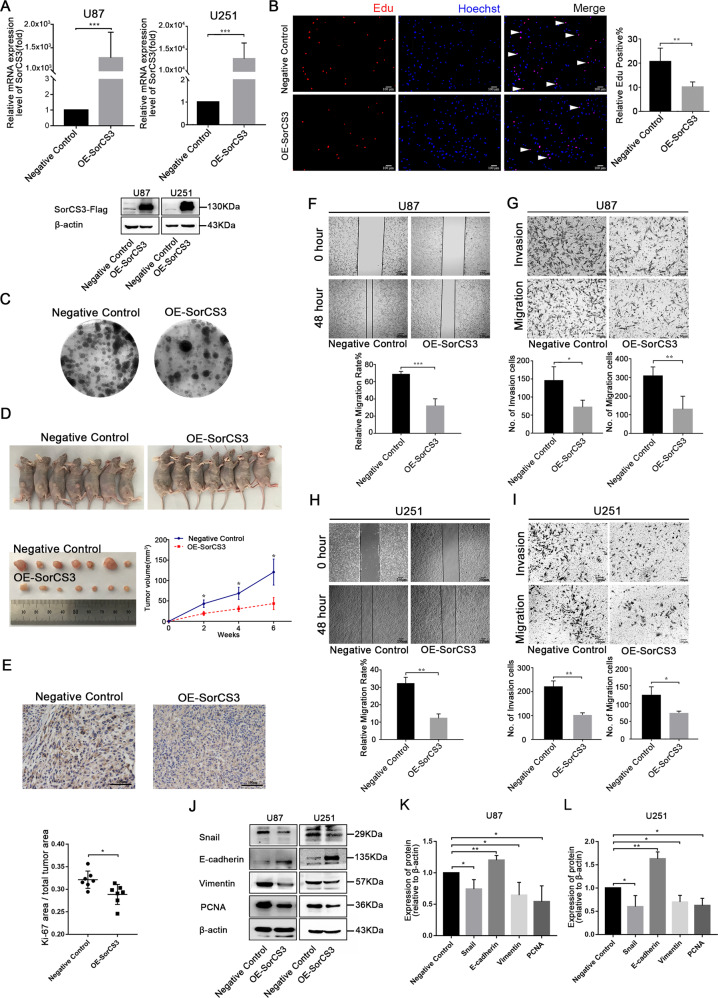
Overexpression of SorCS3 inhibits the migration, invasion, and proliferation of the glioma cells. **A** After transfection with the pCMV3-SorCS3-Flag plasmid, the expression levels of SorCS3 were examined through real-time PCR and Western blotting, using β-actin as an endogenous control. **B** Cell proliferation was determined by EdU staining and EdU incorporation was calculated as EdU+ cells/total cells, quantified by ImageJ. Red was stained for proliferation (EdU + ), blue was stained for nucleus. **C** Colony formation assay for assessing the cell proliferation of SorCS3 overexpression. **D** Image of tumours in nude mice bearing U87 cells treated with overexpression of SorCS3 and control. Tumour volume were measured (*n* = 7). **E** Immunohistochemical staining of Ki-67 expression in xenograft tumour tissues (*n* = 7). **F**, **H** Effect of SorCS3 overexpression on wound healing of U87and U251 cells. **G**, **I** Transwell assays used to determine the influence of SorCS3 overexpression on the migratory and invasive abilities of U87 and U251 cells. **J**–**L** Western blot analysis of proliferation and EMT associated marker after SorCS3 overexpression. Data are shown as mean ± S.D. including three independent experiments. *p < 0.05; **p < 0.01; ***p < 0.001.

To investigate the biological effects of SorCS3 in GBM progression, we first examined cellular SorCS3 levels in a quantitative manner. As shown in Fig. S2A (Western blots were given in Original Data of Supplemental Material.), western blot analysis showed that SorCS3 expression was significantly decreased in the U87 cell line compared to the other glioma cell lines tested. Next, we performed cell proliferation, invasion and migration assays in four glioma cell lines and patient-derived GBM cell lines after transfection with the pCMV3-SorCS3-Flag plasmid (Fig. 2A, C, F, I, Fig. S1B and Fig. S2A–C, Western blots were given in Original Data of Supplemental Material).

SorCS3 overexpression reduced the proliferation of U87 and U251 cells, as determined by an EdU incorporation assay and a plate colony formation assay (Fig. 2B, C). In vivo, overexpression of SorCS3 is associated with inhibited cellular proliferation (Fig. 2D, E). Given that SorCS3 regulates cell proliferation, we examined whether SorCS3 regulates cell cycle and apoptosis. We found that SorCS3 expression in GBM cells decreased the number of cells in G2/S phase but increased the number of cells in G0/G1 phase. However, overexpression of SorCS3 had no effect on apoptosis (Fig. S1C, D). In vitro wound healing and Transwell assays demonstrated that overexpression of SorCS3 reduced the migratory and invasive potential of tumour cells (Fig. 2F–I). These findings showed that SorCS3 significantly attenuates the growth of glioma cells in vitro. Hence, we examined the effects of SorCS3 on the expression of proliferation and EMT markers. Western blot analysis showed that in SorCS3-overexpressing glioma cells, the protein expression of the epithelial marker E-cadherin was upregulated, while the expression of the mesenchymal marker vimentin, as well as the transcriptional repressors Snail and PCNA, was markedly decreased (Fig. 2J–L, Western blots were given in Original Data of Supplemental Material).

**Fig. 3:**
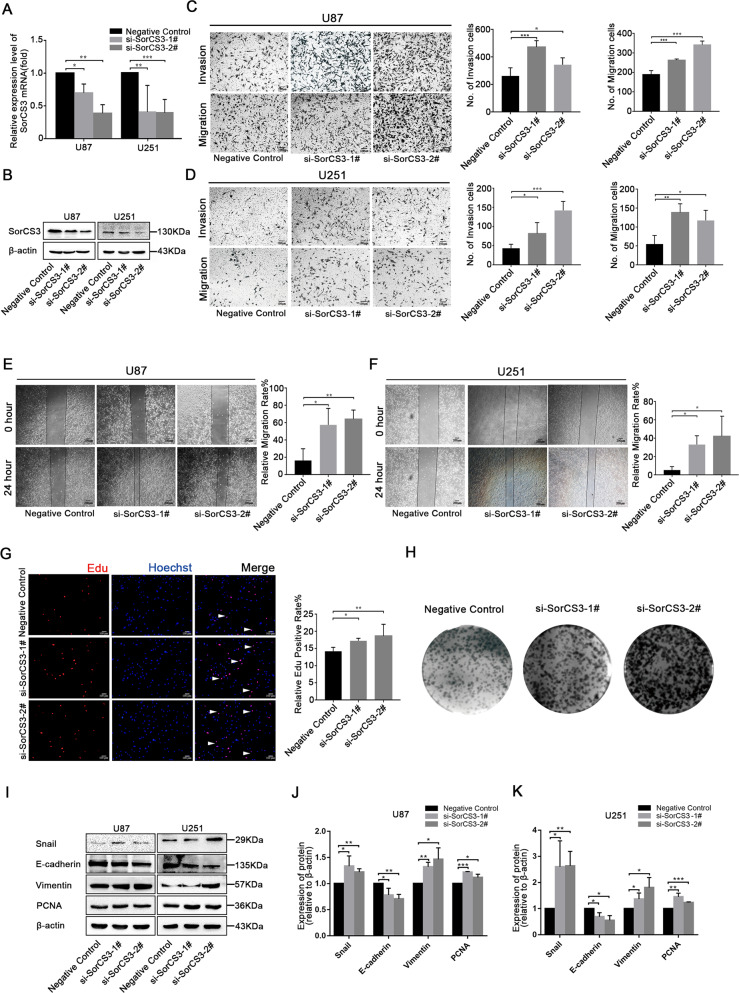
Knockdown of SorCS3 promotes the proliferation, migration and invasion of glioma cells in vitro. **A**, **B** After siRNA-SorCS3-1# and 2# transfection, the expression levels of SorCS3 were examined through real-time PCR and western blotting, using β-actin as an endogenous control. **C**, **D** Transwell assays used to determine the influence of transfection with si-SorCS3-1# and 2# on the migratory and invasive abilities of U87 and U251 cells. **E**, **F** Effect of SorCS3 knockdown on wound healing of U87 and U251 cells. **G** Cell proliferation was determined by EdU staining in SorCS3 knockdown condition and EdU incorporation was calculated as EdU+ cells/total cells, quantified by ImageJ. Red was stained for proliferation (EdU + ), blue was stained for nucleus. **H** Colony formation assay for assessing the cell proliferation of knockdown SorCS3. **I**–**K** Western blot analysis of proliferation- and EMT-associated marker after transfection with SorCS3 siRNA. Data are shown as mean ± S.D. including three independent experiments. *p < 0.05; **p < 0.01; ***p < 0.001.

To verify the tumour suppressor roles of SorCS3, we silenced SorCS3 expression using two siRNA constructs si-SorCS3-1# and 2# (Fig. 3A, B, Western blots were given in Original Data of Supplemental Material). Knockdown of SorCS3 significantly increased the invasion and migration of GBM cells compared to that of control cells in the Transwell assay (Fig. 3C, D). Accordingly, we observed similar patterns in the wound healing assay (Fig. 3E, F). Similarly, we found that knockdown of SorCS3 significantly enhanced the proliferation of GBM cells (Fig. 3G, K, Western blots were given in Original Data of Supplemental Material). In summary, we concluded that SorCS3 functions as a tumour suppressor gene in GBM cells in vitro.

### SorCS3 and p75^NTR^ have a negative regulatory relationship

**Fig. 4:**
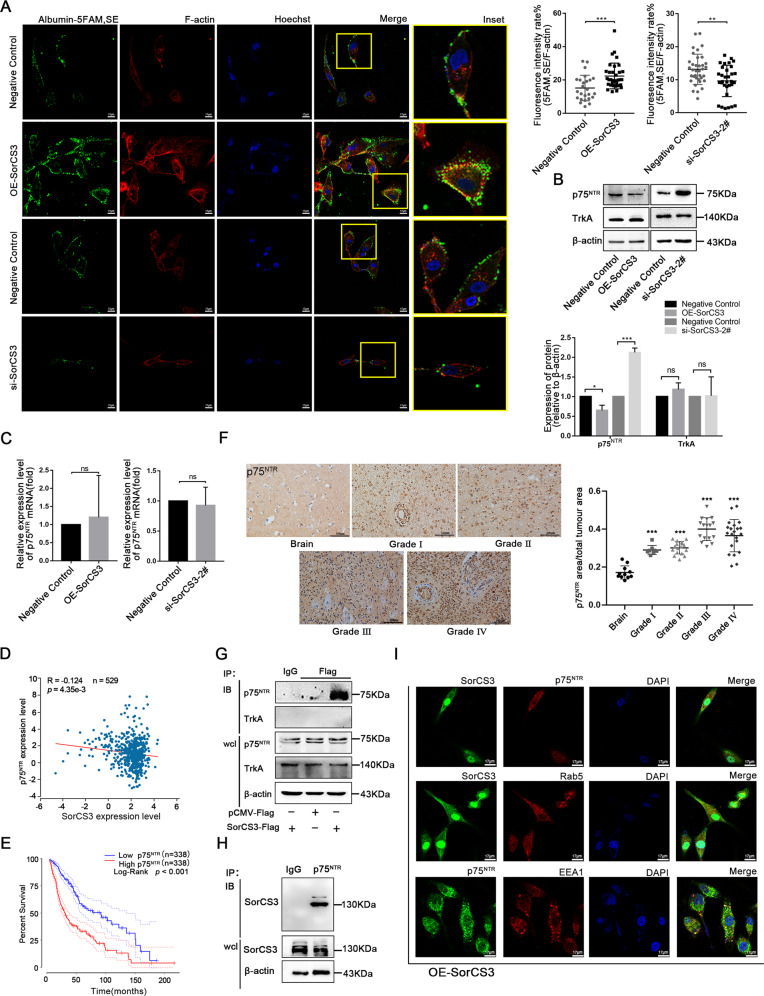
SorCS3 regulates p75^NTR^ expression and internalization. **A** Confocal microscopy images of live cells were treated with Albumin-labeled 5-FAM,SE in altered SorCS3 expression condition. Scale bar: 17 µm. **B**, **C** qPCR and Western blot analysis of p75^NTR^ and TrkA levels after altering the expression of SorCS3 in U87 cells. **D** Correlation between SorCS3 and p75^NTR^ mRNA expression levels were analyzed using starBase v2.0 (*n* = 529, *R* = −0.124, *p* = 4.35e-3). **E** Kaplan-Meier survival curves of glioma from TCGA with different p75^NTR^ expression levels (High SorCS3, *n* = 338; Low SorCS3, *n* = 338; *p* < 0.001). **F** p75^NTR^ expression with different WHO grade glioma and normal brain tissues taken from 108 patients was assessed by IHC. Scale bar: 100 µm. Immunoprecipitations were performed using anti-Flag (**G**) or p75^NTR^ (**H**) antibody, and the immunocomplexes were immunoblotted (IB) using anti-SorCS3, p75^NTR^ and TrkA antibody in transfected SorCS3-Flag plasmid. In parallel, immunoblots for SorCS3, p75^NTR^ and TrkA were performed on whole-cell lysates (wcl); the isotypic lane Immunoglobulin G (IgG) represents the IP control. **I** U87 cells were transfected with SorCS3-Flag plasmid, and then immunolabeled for SorCS3 and markers of the early endosome (Rab5、EEA1) and the p75^NTR^. Scale bar: 17 µm.

The VPS10p domain receptor SorCS3 has been implicated in several pathways of protein internalization and sorting between the plasma membrane and endosomes. Although findings from an earlier report indicate that SorCS3 binds NGF and that NGF is an influencing factor of glioma cell progression [9], the functional implications of these interactions are poorly understood [11]. In order to explore the ability of SorCS3 to induce internalization by specific ligand in GBM cell lines. We evaluated the internalization of SorCS3 by detecting fluorescency labelled albumin in GBM cells. Confocal microscopy revealed that the overexpression of SorCS3 on the cell membrane surface increased the internalization of albumin. Moreover, silencing SorCS3 suppressed the specific ligand-induced internalization ability and decreased the appearance of bright fluorescent puncta on the cell membrane surface (Fig. 4A). Next, we validated the correlation between the SorCS3 and NGFR levels after transfection with the OE-SorCS3 plasmid or SorCS3 siRNA. Our findings showed that the SorCS3 and p75^NTR^ protein levels were negatively related but that the SorCS3 and TrkA levels were not significantly related (Fig. 4B, Western blots were given in Original Data of Supplemental Material). To examine whether the changes in p75^NTR^ protein were caused by changes in the mRNA expressions, we detected mRNA expressions of p75^NTR^ by using qPCR. qPCR results showing that overexpression or depletion of SorCS3 did not affect the mRNA expression of p75^NTR^ (Fig. 4C). Therefore, we analysed the correlation between the mRNA levels of SorCS3 and p75^NTR^ in TCGA. Interestingly, the results showed a weak correlation between these levels (Fig. 4D). We verified the biological significance of p75^NTR^ in GBM. Kaplan-Meier analysis indicated that high expression of p75^NTR^ was negatively correlated with the overall survival time of GBM patients (Fig. 4E). Further, we examined the expression of p75^NTR^ in glioma samples of different grades by tissue microarray analysis. Statistical chart showed the expression level of p75^NTR^ in the different grades of glioma tissues. (Fig. 4F). Taken together, these data indicated that SorCS3 regulates the expression of p75^NTR^ in GBM.

### SorCS3 interacts with p75^NTR^ on the plasma membrane

Intrigued by the co-internalization of SorCS3 and p75^NTR^, we generated a SorCS3-Flag fusion protein and then performed a set of Co-IP assays to investigate whether SorCS3 and p75^NTR^ interact. As shown in Fig. 4G, SorCS3 and p75^NTR^ may interact. However, there seemed to be no interaction between SorCS3 and TrkA. Next, we found that endogenous p75^NTR^ and SorCS3 co-precipitated in U87 cells, indicating that SorCS3 and p75^NTR^ may exist in the same protein complex (Fig. 4H, Western blots were given in Original Data of Supplemental Material). In GBM cells, the overexpressed SorCS3 partial co-localization with p75^NTR^ and endosomal markers Rab5 and EEA1 (Fig. 4I). We speculated that p75^NTR^ may be involved in the internalization process regulated by SorCS3. Taken together, these findings indicated that SorCS3 co-precipitates with p75^NTR^.

### NGF stimulation promotes the interaction between SorCS3 and p75^NTR^

**Fig. 5:**
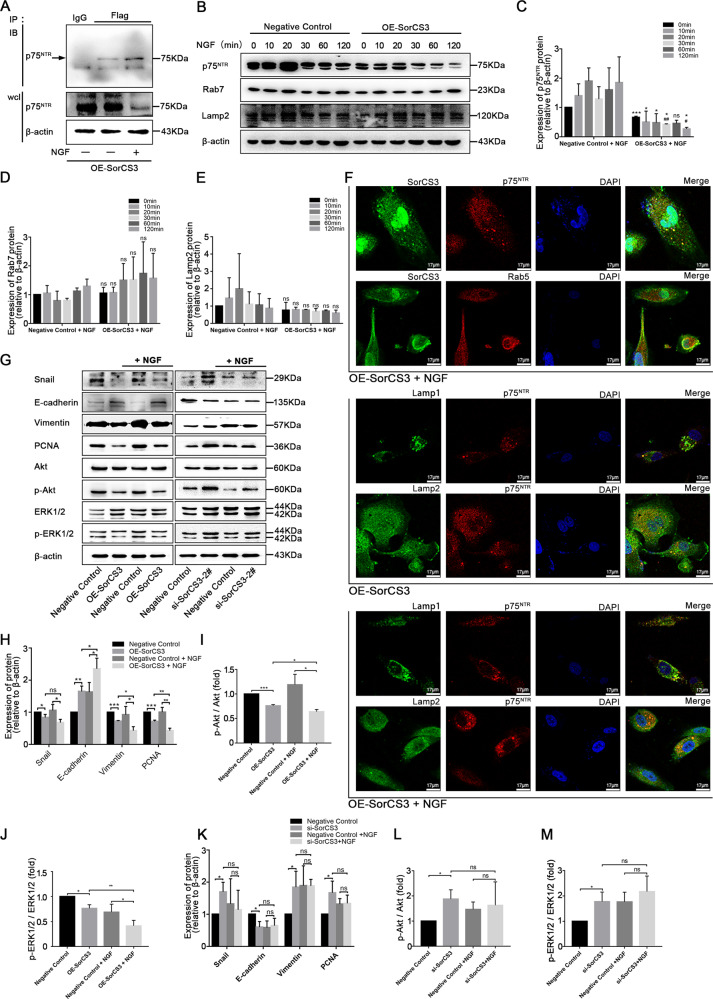
NGF promotes the SorCS3 and p75^NTR^ interaction. **A** U87 cells were transfected SorCS3-Flag plasmid, and then stimulated or not with NGF (100 ng/mL) for 60 min. IP were performed using anti-Flag antibody, and the immunocomplexes were immunoblotted (IB) using anti-p75^NTR^ antibody. In parallel, immunoblots for p75^NTR^ were performed on whole-cell lysates (wcl); the isotypic lane Immunoglobulin G (IgG) represents the IP control. **B**–**E** U87 overexpression of SorCS3 and control were stimulated with NGF (100 ng/mL) over 0–120 min time course. Cell lysates were analyzed by western blotting for components of the canonical internalization signaling pathway using the indicated antibodies. *p vs. the control group, #*p* vs. the OE-SorCS3 (NGF, 0 min) group. ns: not statistically significant. **F** Process of internalization was visualized by immunofluorescence staining of the endosome and the lysosomal marker. U87 cells were stimulated with NGF (100 ng/mL) for 60 min, and then immunofluorescence for SorCS3-Flag, p75^NTR^, early endosome markers (Rab5) and lysosome markers (Lamp1 and Lamp2). Scale bar: 17 µm. **G**–**M** U87 were transfected with SorCS3-Flag plasmid or siRNA (si-SorCS3-2#) and the corresponding control, and then stimulated with NGF (100 ng/mL) for 60 min. Cell lysates were analyzed by western blotting with the indicated antibodies, using β-actin as an endogenous control. Data are shown as mean ± S.D. including three independent experiments. *p < 0.05; **p < 0.01; ***p < 0.001.

We then sought to investigate the mechanism underlying the SorCS3-p75^NTR^ interaction. We stimulated U87 cells with NGF under normal conditions, investigated whether NGF stimulation promoted the interaction between p75^NTR^ and SorCS3, and analysed the canonical internalization pathways of SorCS3 in whole-cell lysates (wcl). In addition, the interaction between p75^NTR^ and SorCS3 increases with NGF treatment (Fig. 5A, Western blots were given in Original Data of Supplemental Material). Interestingly, in cells with SorCS3 overexpression, the expression of p75^NTR^ gradually decreased with increasing NGF treatment time. Interestingly, the expression level of the lysosomal marker Lamp2 and the late endosome marker Rab7 was not changed (Fig. 5B–E, Western blots were given in Original Data of Supplemental Material). Usually, receptor internalization is often involved in lysosomal trafficking and degradation. Hence, we speculated that the lysosome degradation pathway might be involved in p75^NTR^ degradation. Under NGF with or without treatment conditions, confocal immunofluorescence microscopy was performed for SorCS3, p75^NTR^, lysosomal marker and the endosome marker subcellular localization. Immunofluorescence experiments showed that the localization of these punctate structures (p75^NTR^) overlapped with lysosomes (Lamp1/Lamp2) was increased (Fig. 5F). Overexpression of SorCS3 impaired p75^NTR^ signalling, as evidenced by the decreased phosphorylation of both Akt and ERK1/2. After treatment with NGF, the inhibitory effect on the p75^NTR^ signal was enhanced to some extent. In addition, SorCS3-depleted cells exhibited sustained p75^NTR^ signalling (Fig. 5G–M, Western blots were given in Original Data of Supplemental Material). Taken together, these results suggested that SorCS3 can weaken the cancer-promoting effect of NGF and that NGF can enhance the SorCS3-regulated internalization of p75^NTR^.

### Inhibition of internalization abolishes the tumour-suppressive ability of SorCS3

**Fig. 6:**
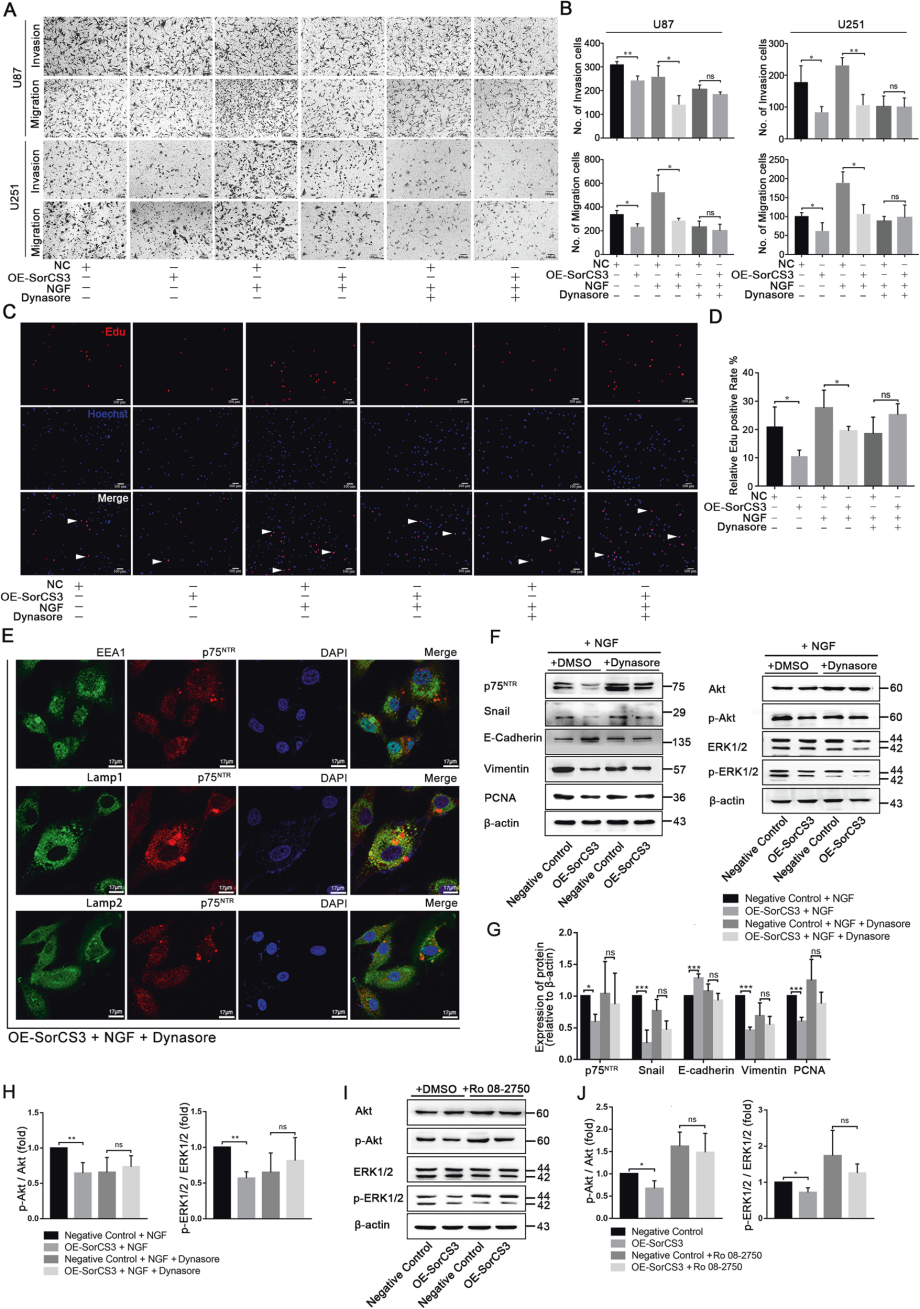
Inhibition of internalize abolishes the tumour suppressor effects of SorCS3. **A**, **B** Transwell assays were used to determine the influence of U87 and U251 cells pretreated with or without the cell-permeable dynamin inhibitor Dynasore (40 µM) for 2 h and then stimulated with NGF (100 ng/mL) for 60 min in overexpression of SorCS3 condition. **C**, **D** EdU staining to determine the influence of SorCS3 overexpression pretreated with or without the cell-permeable dynamin inhibitor Dynasore (40 µM) for 2 h and then stimulated with NGF (100 ng/mL) for 60 min, and EdU incorporation was calculated as EdU+ cells/total cells, quantified by ImageJ. Red was stained for proliferation (EdU + ), blue was stained for nucleus. **E** U87 cells were stimulated with Dynasore (40 µM) for 2 h and then stimulated with NGF (100 ng/mL) for 60 min, and then immunofluorescence for p75^NTR^, early endosome markers (EEA1) and lysosome markers (Lamp1 and Lamp2). Scale bar: 17 µm. **F**–**H** U87 were pretreated with the cell-permeable dynamin inhibitor Dynasore (40 µM) for 2 h and then stimulated with NGF (100 ng/mL) for 60 min in overexpression of SorCS3 condition. Cell lysates were analyzed by western blotting with the indicated antibodies, using β-actin as an endogenous control. **I**, **J** U87 were pretreated with the Ro 08-2750 (1 µM, Ro 08-2750 is a reversible NGF inhibitor which inhibits NGF binding to p75^NTR^ selectively) for 8 h in overexpression of SorCS3 condition. Cell lysates were analyzed by western blotting with the indicated antibodies, using β-actin as an endogenous control. Data are shown as mean ± S.D. including three independent experiments. ns: not statistically significant.

To verify the role of NGF in SorCS3-mediated inhibition of GBM, we analysed the biological functions of proliferation, invasion and migration in SorCS3 overexpressing GBM cells treated with NGF (Fig. 6A, D). Under normal conditions, when SorCS3 is overexpressed, it inhibits the invasion and migration of GBM cells. However, after NGF treatment, the effect of SorCS3 was amplified in two glioma cell lines (U87 and U251). Then, we inhibited internalization using Dynasore, we also added NGF to promote internalization mediated by SorCS3. In Dynasore-treated cells, the inhibitory effect of SorCS3 on migration and invasion was abolished (Fig. 6A, B). As expected, in the EdU incorporation assay, the number of proliferating cells did not change significantly (Fig. 6C, D). Dynasore treatment decreases colocalization of the p75^NTR^ with EEA1, Lamp1 and Lamp2, which abolished the SorCS3 mediated internalization effect (Fig. 6E). Subsequently, we investigated whether suppression of internalization impairs the SorCS3-p75^NTR^ signalling pathway. Interestingly, in cells with SorCS3 overexpression, the decrease in the p75^NTR^ level was alleviated by the inhibition of internalization. Blockade of internalization suppressed the change in p75^NTR^ signalling, as evidenced by the unchanged phosphorylation of both AKT and ERK1/2 (Fig. 6F–H, Western blots were given in Original Data of Supplemental Material). To further verify that the biological function of SorCS3 in suppressing cancer is achieved through the NGF/p75^NTR^ signalling pathway, we selected the competitive NGF inhibitor Ro 08-2750 to inhibit binding between endogenous NGF and p75^NTR^. As a result, we found that Ro 08-2750 restored the influence of SorCS3 on the downstream p75^NTR^ signalling pathway (Fig. 6I, J, Western blots were given in Original Data of Supplemental Material). Taken together, these findings indicated that inhibiting the internalization or the binding between NGF and SorCS3 interfered with the tumour suppressor function of SorCS3.

**Fig. 7:**
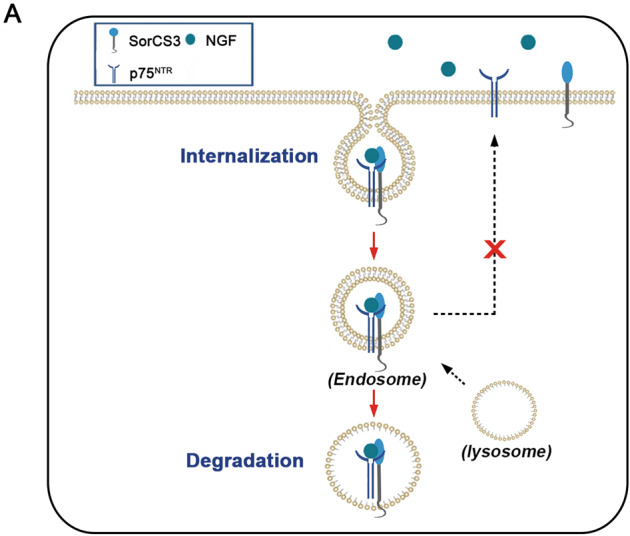
SorCS3 expression decreased with the tumour aggressiveness. **A** SorCS3 exerts tumour suppressor effect by enhance p75^NTR^ internalization and transport to lysosome degradation in glioma.

In this study, we demonstrate that SorCS3, a sorting protein previously not investigated in carcinomas, is expressed at low levels in GBM cell lines and patients. In this model, SorCS3 regulates rapid internalization of p75^NTR^ by forming a complex with this receptor and coupling it to lysosomal degradation, limiting invasion and proliferation signalling via p75^NTR^ (Fig. 7A).

## Discussion

SorCS3 is a type I transmembrane protein that mediates the signalling and/or intermembrane transport of ligands and is regarded as a sorting receptor with important neuronal functions. Previous studies have shown that SorCS3 is exclusively expressed in the brain with tissue specificity. However, we detected the expression of SorCS3 in glioma tissue microarray samples and cell lines (Fig. 1C, D and Fig. S1A). In addition, we analysed data from the TCGA database and found that the expression of SorCS3 in gliomas is significantly reduced. The molecular characteristics of glioma classification are crucial for the treatment and prognosis of glioma. In the CGGA dataset, we evaluated the correlations between SorCS3 expression and histological type, clinical factors, and molecular characteristics. Low expression of SorCS3 was closely related to IDH wild-type gliomas, 1p/19q gliomas, and recurrent gliomas (Fig. S3A–F). However, there was no association with sex (Fig. S3G). These findings indicate that SorCS3 may have important biological functions in GBM.

The oncogenic and tumor-suppressive functions of molecules likely depend on the mechanism involved [12]. According to the biological characteristics of SorCS3, which may be involved in the process of proliferation and invasion signalling pathways, SorCS3 acts as a regulator of cell membrane receptor internalization. Abnormal signal transduction is one of the main characteristics of tumour progression. It is the result of cooperation among multiple driving factors. In addition to gene mutation, epigenetic modification, and transcriptome regulation, internalization directly leads to receptor degradation or recycling to regulate the activation of signalling pathways. For example, Fabrice et al. found that Sortilin is a regulator of EGFR intracellular trafficking that promotes receptor internalization and limits signalling, which in turn impacts tumour growth [13]. Some studies have shown that SorLA, through interactions at its extracellular domain, exists in a complex with HER2 and co-traffics with HER2, facilitating HER2 recycling to the plasma membrane to support HER2 downstream signalling. In the absence of SorLA, HER2 becomes localized to enlarged, partially dysfunctional lysosomes, resulting in defective HER2 signalling and increased sensitivity [14, 15]. Increasing evidence indicating the important roles that Sortilin and SorLA participate in a variety of tumour progression-related processes. SorCS3, Sortilin and SorLA are members of the same VPS10p family; they have similar biological functions and are highly conserved among different species. We found that SorCS3-mediated cellular internalization is one of the key mechanisms for suppressing glioma metastasis and proliferation.

NGF, by binding to two specific membrane receptors, TrkA and p75^NTR^, activates different downstream signalling cascades [16]. SorCS3 is a NGF receptor and can bind to NGF [11]. However, the relationship between SorCS3 and the other two NGFRs has not been investigated. It is not known whether these receptors act independently in competitively binding to NGF or bind to each other to form a complex. We attempted to verify the interaction between SorCS3 and p75^NTR^ using co-immunoprecipitation assays and found that this interaction might exist (Fig. 4H). In glioma, p75^NTR^ has been reported to be an important regulator of cell viability, invasion, and migration [17–19]. For example, p75^NTR^ regulates invasion and progression through γ-secretase-dependent and γ-secretase-independent mechanisms in glioma [20, 21]. Further, phosphorylation of p75^NTR^ on S303 and S425 is necessary for glial tumour invasion [22].

p75^NTR^ can delay the internalization and degradation of TrkA after NGF treatment to promote the potentiation of TrkA signalling [23]. Our studies showed that SorCS3 interacts with p75^NTR^. By immunofluorescence, p75^NTR^ was found to be internalized via cell membrane receptor-mediated internalization. However, at this point, we do not know whether SorCS3 interacts directly with p75^NTR^ or whether they are coupled through a molecular complex or an intermediary partner. We speculate that NGF may act as a bridge that links the SorCS3 and p75^NTR^ proteins, since the SorCS3-p75^NTR^ interaction was markedly increased by NGF treatment. In addition, other research has shown that proNGF can form a ternary complex with Sortilin or SorCS2 and p75^NTR^ as co-receptors, which do not interact directly but are separated by the proNGF dimer, which acts as a separating wedge [24, 25]. This is very similar to the predicted SorCS3-p75^NTR^ interaction model (Fig. S4).

In our study, NGF treatment accelerated the routing of p75^NTR^ towards rapid internalization and degradation by SorCS3 (Fig. 5B). Many classic studies have shown that once internalized from the plasma membrane, membrane-bound vesicles that carry receptors (VPS10p family members) from the cell surface fuse with the early endosome. The early endosome (EE) serves as a sorting station from which cargoes are either delivered to the endo-lysosomal system for degradation or are recycled directly or indirectly to the plasma membrane via the endocytic recycling compartment; endocytic recycling is primarily a default pathway whereby proteins, in the absence of selective signals to target them for degradation, are typically recycled [26]. For example, VPS10p family members can transport many interacting proteins to the plasma membrane via the endocytic pathway and the lysosome for degradation [27–29]. In addition, sphingolipid activator proteins, acid sphingomyelinase, and cathepsin D and H have been shown to be trafficked by Sortilin to the lysosome [30, 31]. Receptor internalization and subsequent trafficking to the lysosome promotes protein degradation and is a key biological process in terminating signal transduction [32].

However, this study has several limitations. First, blocking internalization with Dynasore did not completely abolish SorCS3-induced p75^NTR^ degradation (Fig. 6F, G). This failure is possibly due to other alternative degradation pathways involved in the effects of SorCS3, including autophagy, ubiquitination and proteasomal degradation pathways. Second, long-term cell culture is susceptible to genetic drift, which can lead to altered phenotypes and growth patterns [33, 34]. Low-passage GBM models should be employed to circumvent these effects. Regrettably, this study did not generate a low-passage GBM model and a representative intracranial assessments model to accurately recapitulate the main features of GBM. Third, we were unable to demonstrate that SorCS3 expression is a reliable prognostic factor for the recurrence and metastasis of GBM. Although extensive studies have been performed to investigate the mechanism of SorCS3 in neurological-associated pathways, the SorCS3-mediated mechanism underlying GBM pathogenesis remains unclear. The findings of this study may facilitate to the development of novel therapeutic strategies for GBM.

## Availability of data and materials

All data generated or analysed during this study are included within the article or available from the corresponding author on reasonable request.

## Supplementary information

Supplemental Figure 1

Supplemental Figure 2

Supplemental Figure 3

Supplemental Figure 4

Supplemental Figure 5

aj-checklist

Original data of WB
