# Supplementary figures and images for: SorCS3 promotes the internalization of p75NTR to inhibit GBM progression
Source: Cell Death Dis. 2022 Apr 7;13(4):313. doi: 10.1038/s41419-022-04753-5 (PMC8989992; doi:10.1038/s41419-022-04753-5)

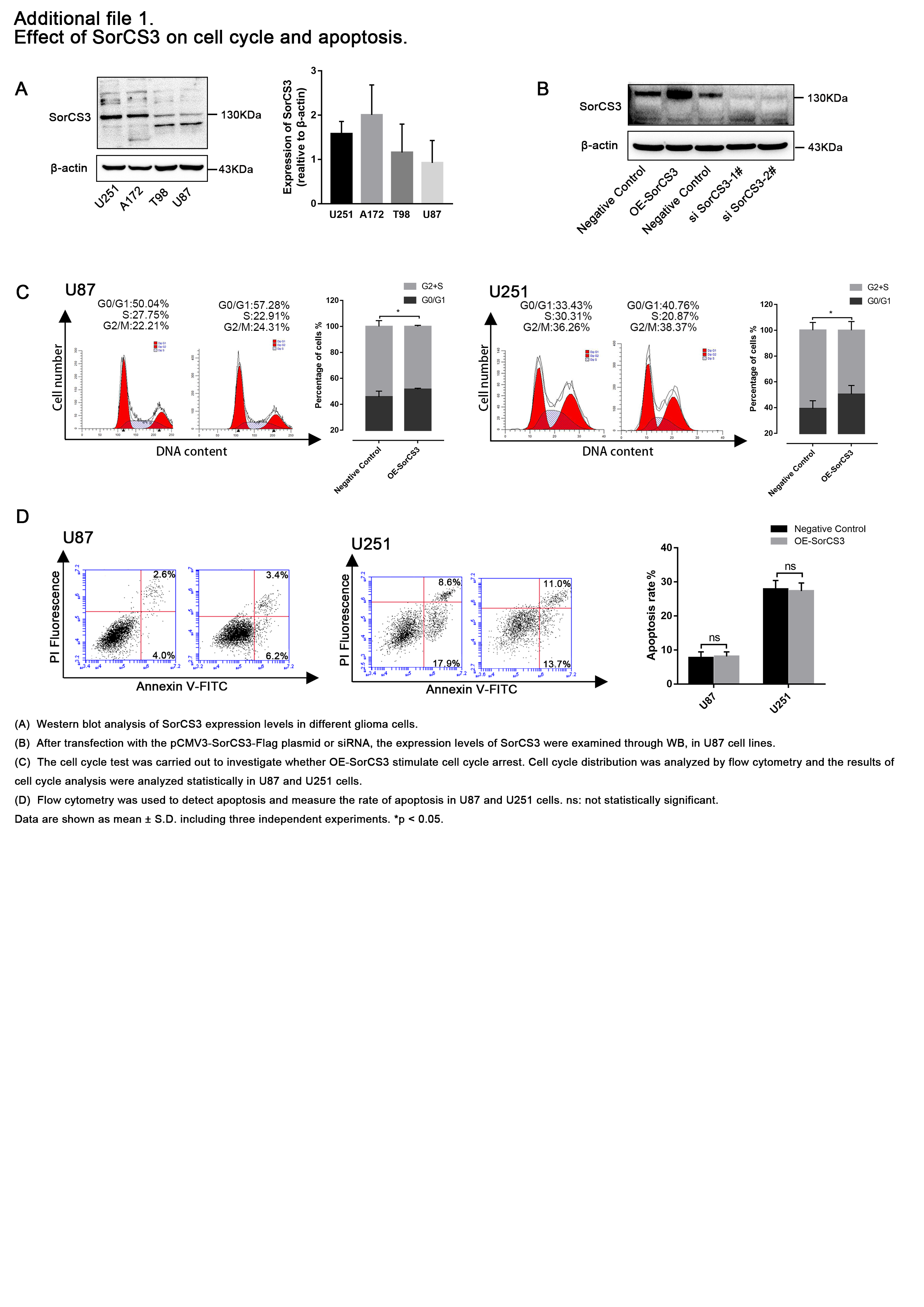

Supplement: Supplementary file 1 — Supplemental Figure 1 [file 41419_2022_4753_MOESM1_ESM.tif]

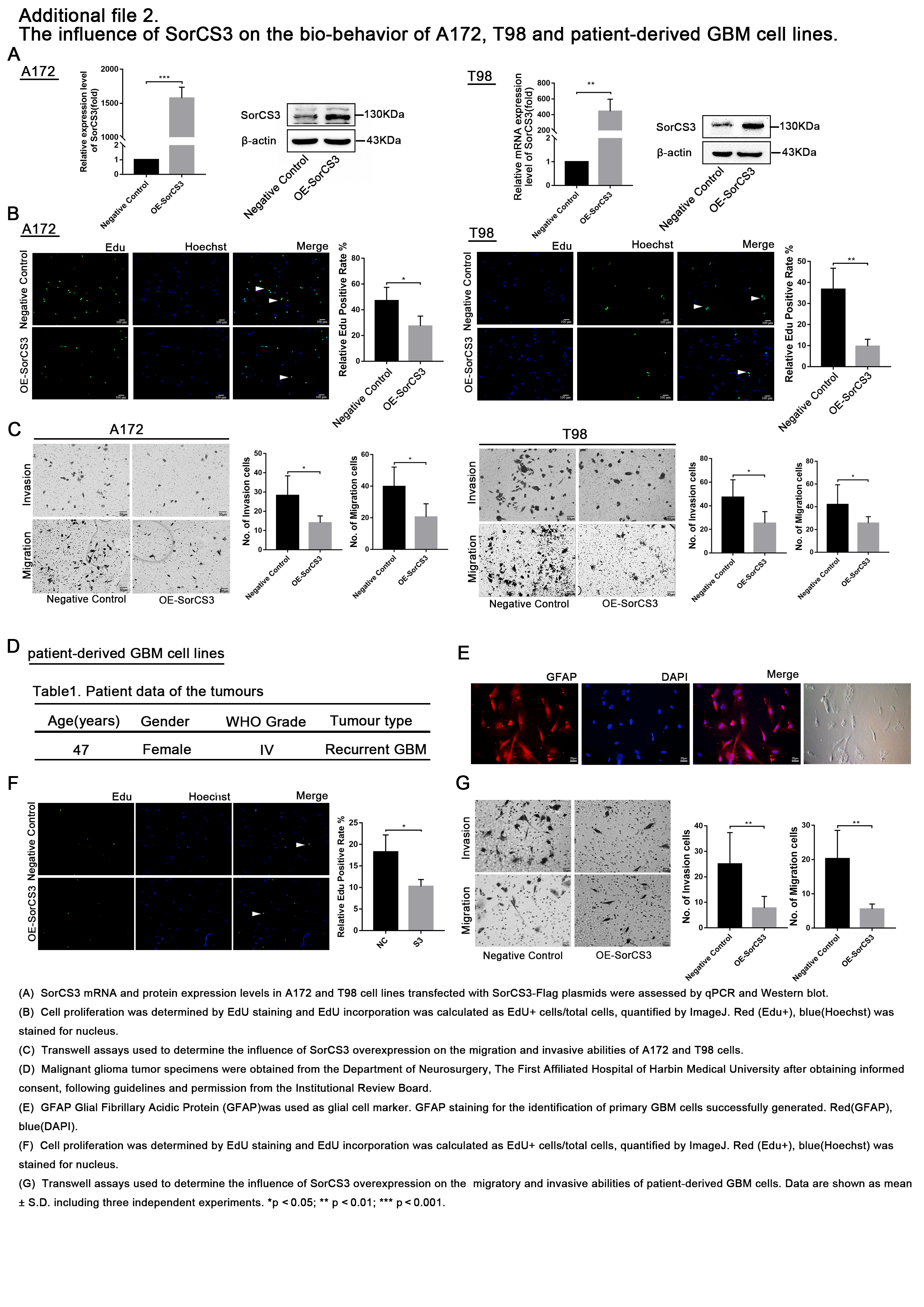

Supplement: Supplementary file 2 — Supplemental Figure 2 [file 41419_2022_4753_MOESM2_ESM.tif]

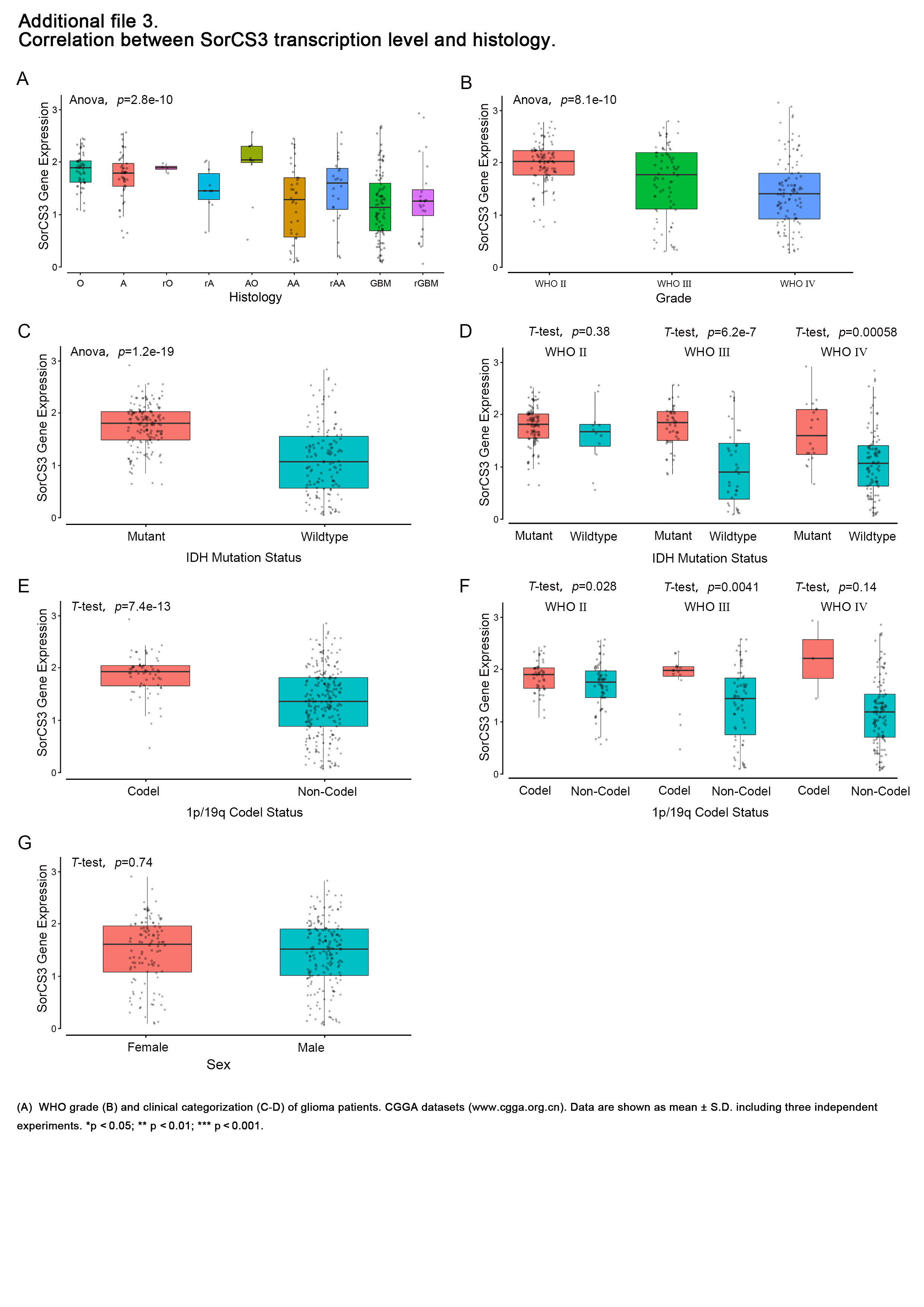

Supplement: Supplementary file 3 — Supplemental Figure 3 [file 41419_2022_4753_MOESM3_ESM.tif]

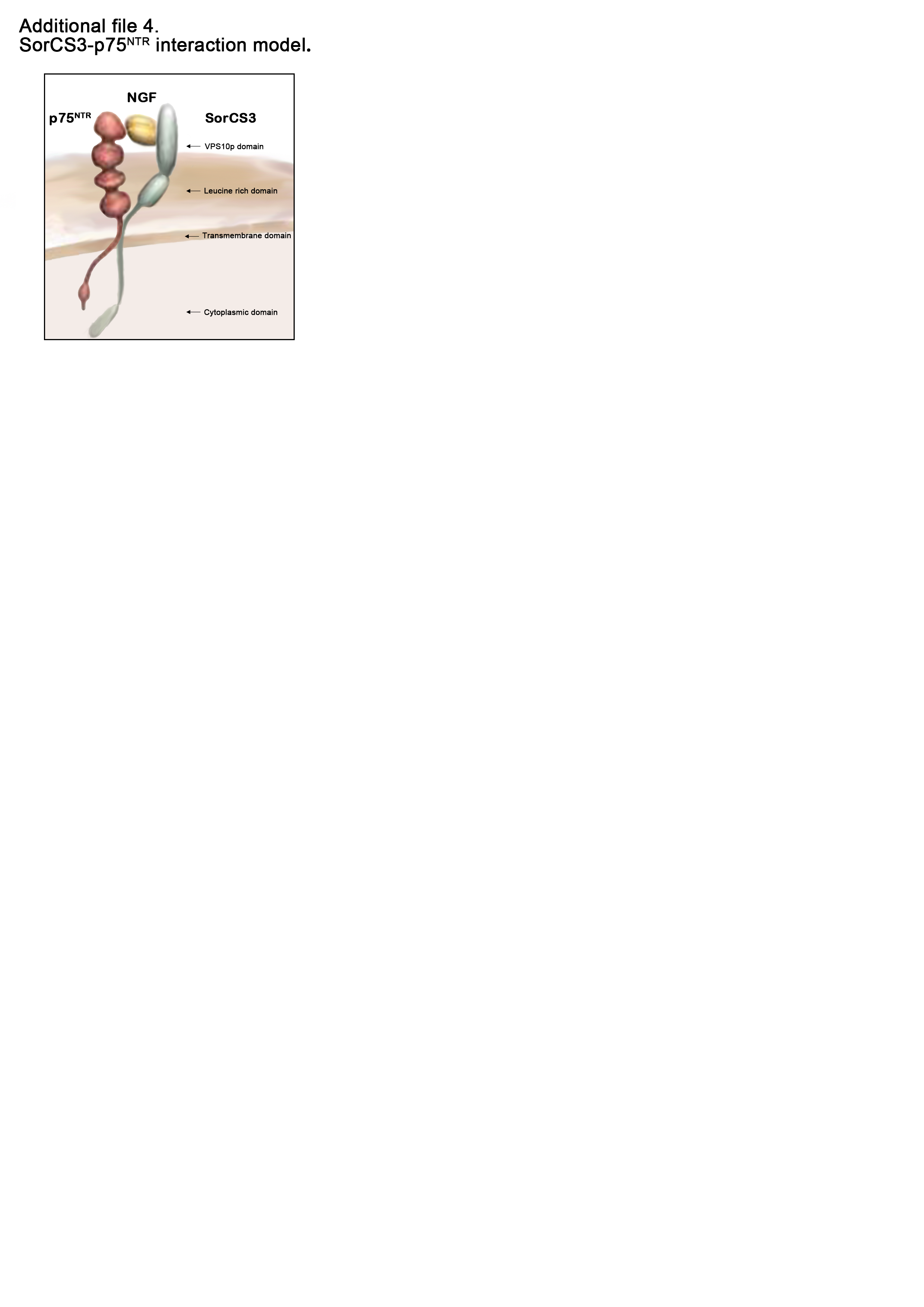

Supplement: Supplementary file 4 — Supplemental Figure 4 [file 41419_2022_4753_MOESM4_ESM.tif]
