# Supplementary material for: SorCS3 promotes the internalization of p75NTR to inhibit GBM progression
Source: Cell Death Dis. 2022 Apr 7;13(4):313. doi: 10.1038/s41419-022-04753-5 (PMC8989992; doi:10.1038/s41419-022-04753-5)
Supplement: Supplementary file 5 — Supplemental Figure 5 [file 41419_2022_4753_MOESM5_ESM.pdf]

Table 2: Information of antibodies used in this study

Antibody Name	Company	Application	Dilution	Host
**SorCS3**	R&D bio-techne	WB	1:1000	Goat
	Antibodies-Online	WB	1:500	Rabbit
	Bioss	WB	1:1000	Rabbit
**p75NTR**	Cell Signaling Technolog (CST)	WB	1:1000	Mouse
		Co-IP	1:50	Rabbit
		IF	1:600	Rabbit
**Akt**	CST	WB	1:1000	Rabbit
**p-Akt** (Ser473)	CST	WB	1:2000	Rabbit
**ERK**	CST	WB	1:1000	Rabbit
**p-ERK** (Thr202/Tyr204)	CST	WB	1:1000	Rabbit
**PCNA**	CST	WB	1:1000	Rabbit
**Snail**	ABcolonal	WB	1:1000	Rabbit
**E-cadherin**	CST	WB	1:1000	Rabbit
**Vimentin**	Wanleibio	WB	1:1000	Rabbit
**TrkA**	CST	WB	1:1000	Rabbit
**β-actin**	ProteinTech	WB	1:1000	Rabbit
**Flag**	ProteinTech	WB	1:1000	Rabbit
	Abcam	Co-IP	1:100	Rabbit
	CST	IF	1:100	Mouse
	ProteinTech	IF	1:100	Rabbit
**Rab7**	ProteinTech	WB	1:1000	Rabbit
**Rab5**	CST	IF	1:100	Rabbit
**EEA1**	ProteinTech	IF	1:100	Mouse
**LAMP2**	ProteinTech	WB	1:500	Rabbit
		IF	1:100	Mouse
**LAMP1**	CST	IF	1:200	Mouse
**GFAP**	ABcolonal	IF	1:100	Rabbit
