# Supplementary material for: SorCS3 promotes the internalization of p75NTR to inhibit GBM progression
Source: Cell Death Dis. 2022 Apr 7;13(4):313. doi: 10.1038/s41419-022-04753-5 (PMC8989992; doi:10.1038/s41419-022-04753-5)
Supplement: Supplementary file 7 — Original data of WB [file 41419_2022_4753_MOESM7_ESM.pdf]

The original data of Western blots

Figure2-A

U87MG

Lane: 1 2 1: NC 2: SorSC3-Flag

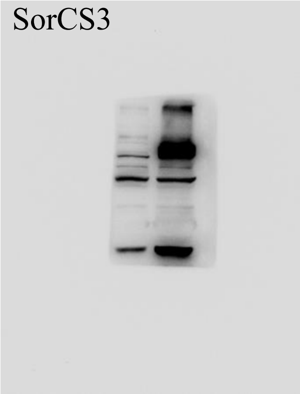

130KDa

Lane: 1 2 1: NC 2: SorSC3-Flag

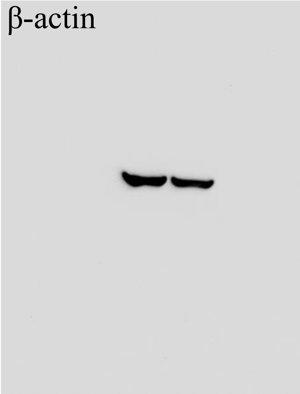

43KDa

U251

Lane: 1 2 3 4 5 6 7 8

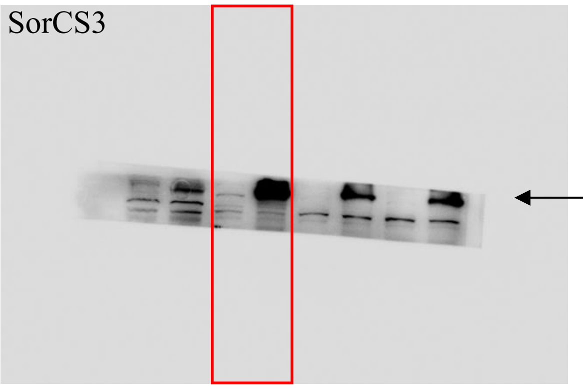

130KDa

Lane: 1 2 3 4 5 6 7 8

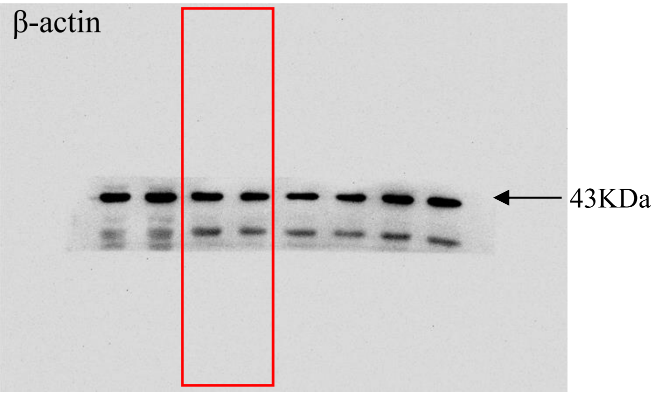

43KDa

Lane

1: NC 2: SorSC3-Flag 3: NC 4: SorSC3-Flag 5: NC 6: SorSC3-Flag 7: NC 8: SorSC3-Flag

Figure2-J

U87MG

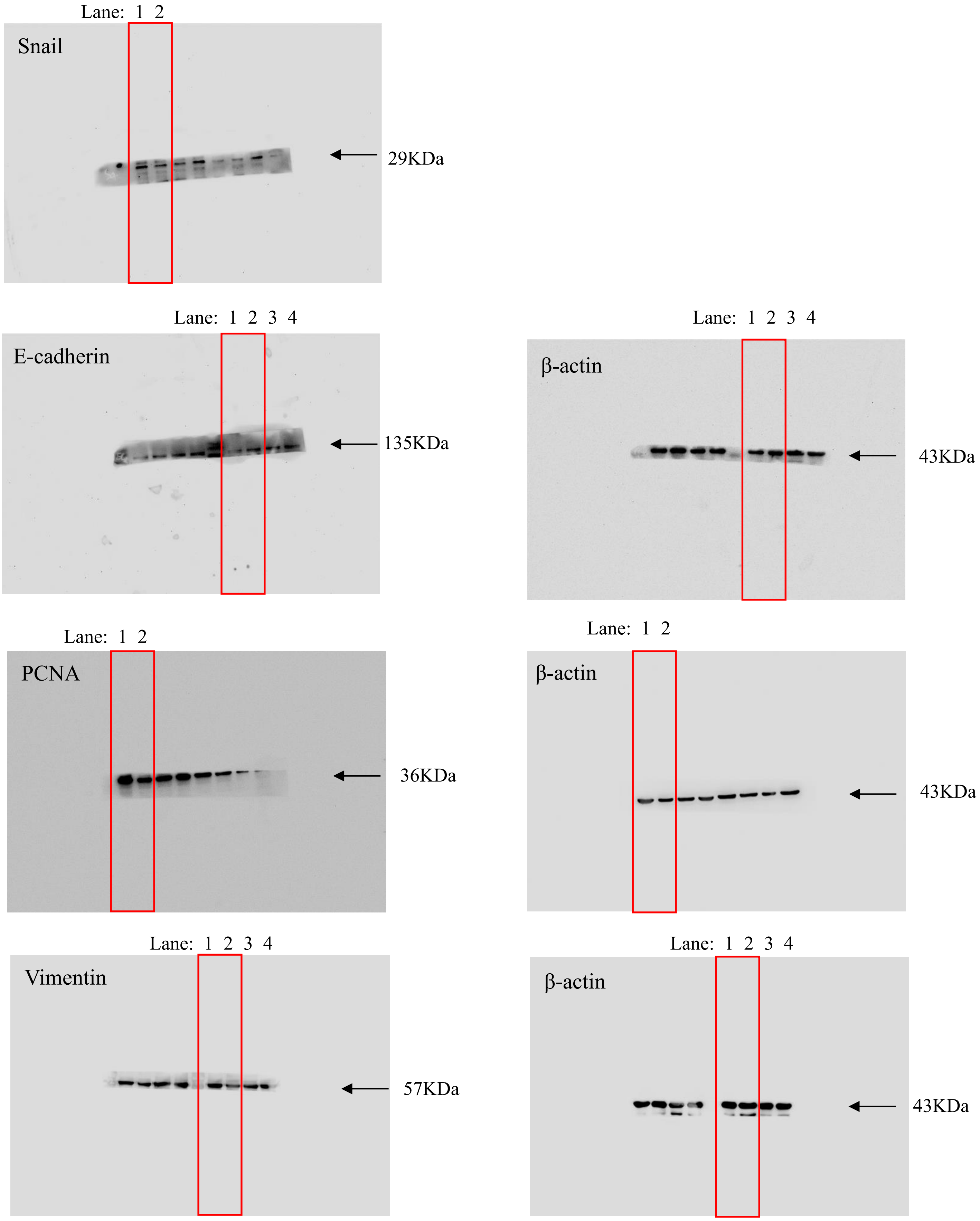

Lane: 1: NC 2: SorSC3-Flag

Figure2-J

U251

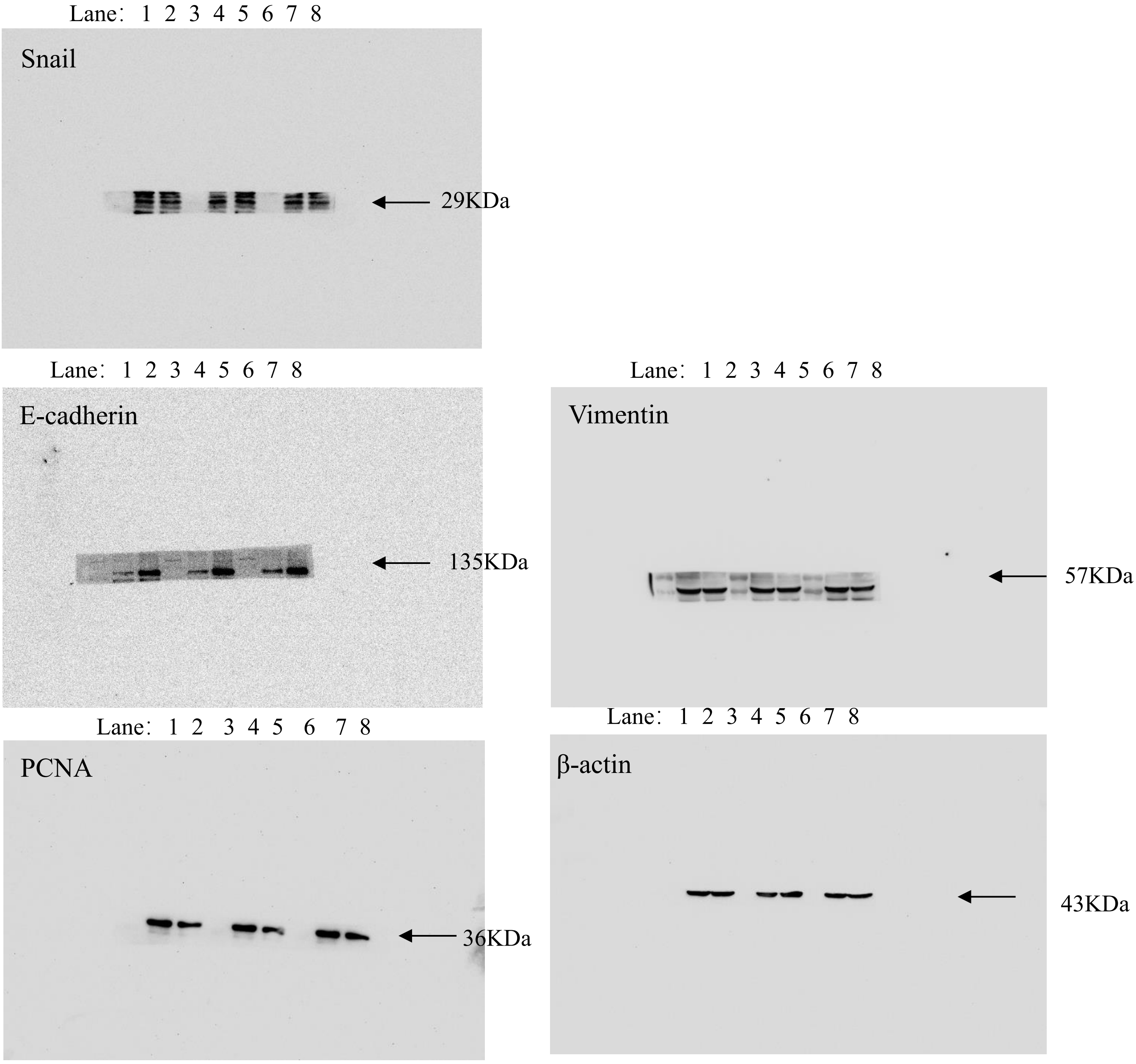

Lane

1: NC 2: OE-SorSC3 3: Marker 4: NC 5: OE-SorSC3 6: Marker 7: NC 8: OE-SorSC3

Figure3-B

U87MG

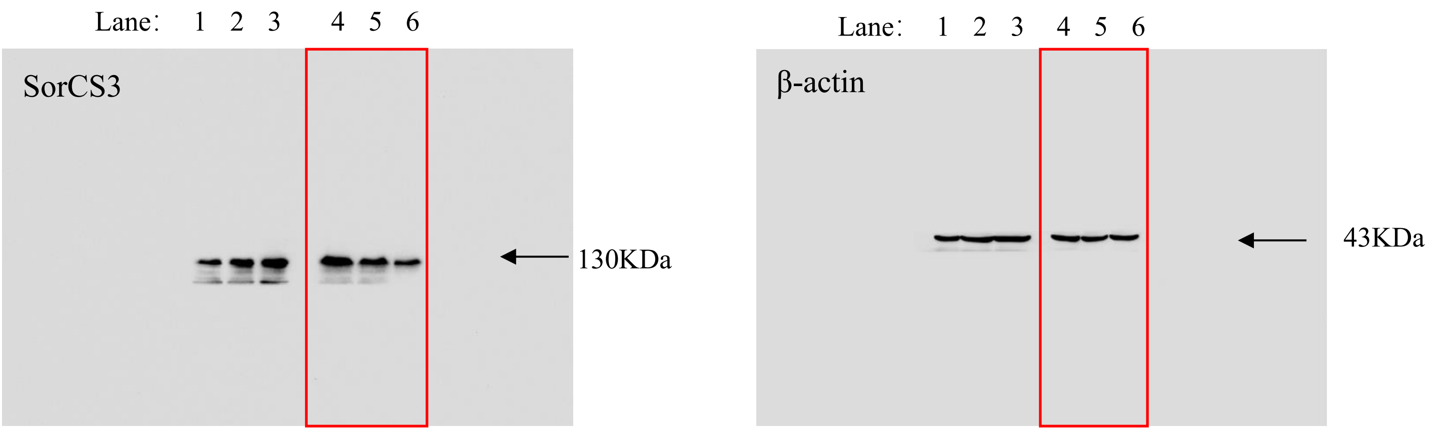

Lane

1: si-SorCS3-2# 2: si-SorCS3-1# 3: NC 4: NC 5: si-SorCS3-1# 6: si-SorCS3-2#

U251

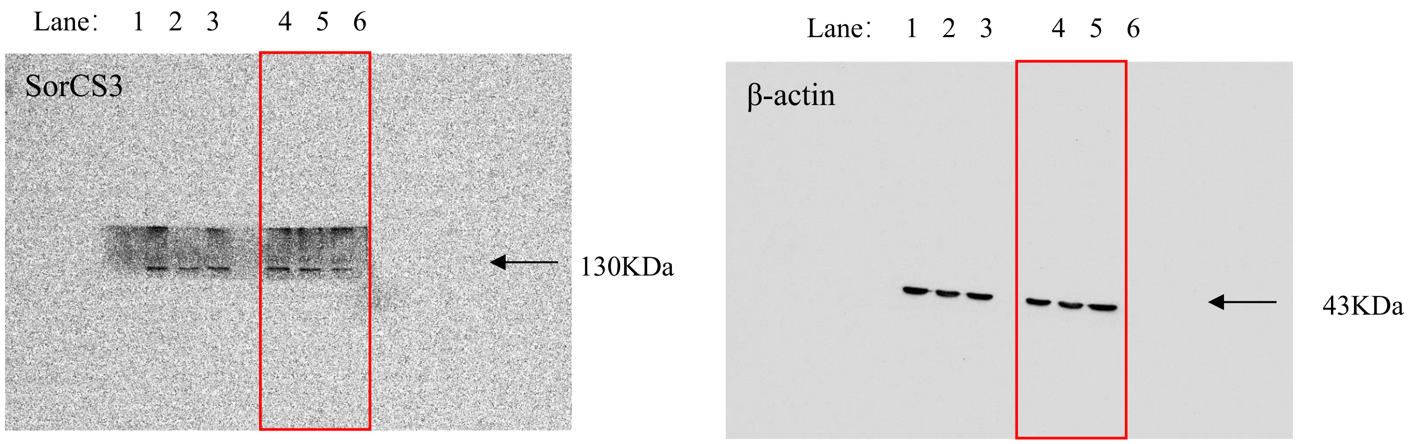

Lane

1 NC 2: si-SorCS3-1# 3: si-SorCS3-2# 4: NC 5: si-SorCS3-1# 6: si-SorCS3-2#

Figure3-I

U87MG

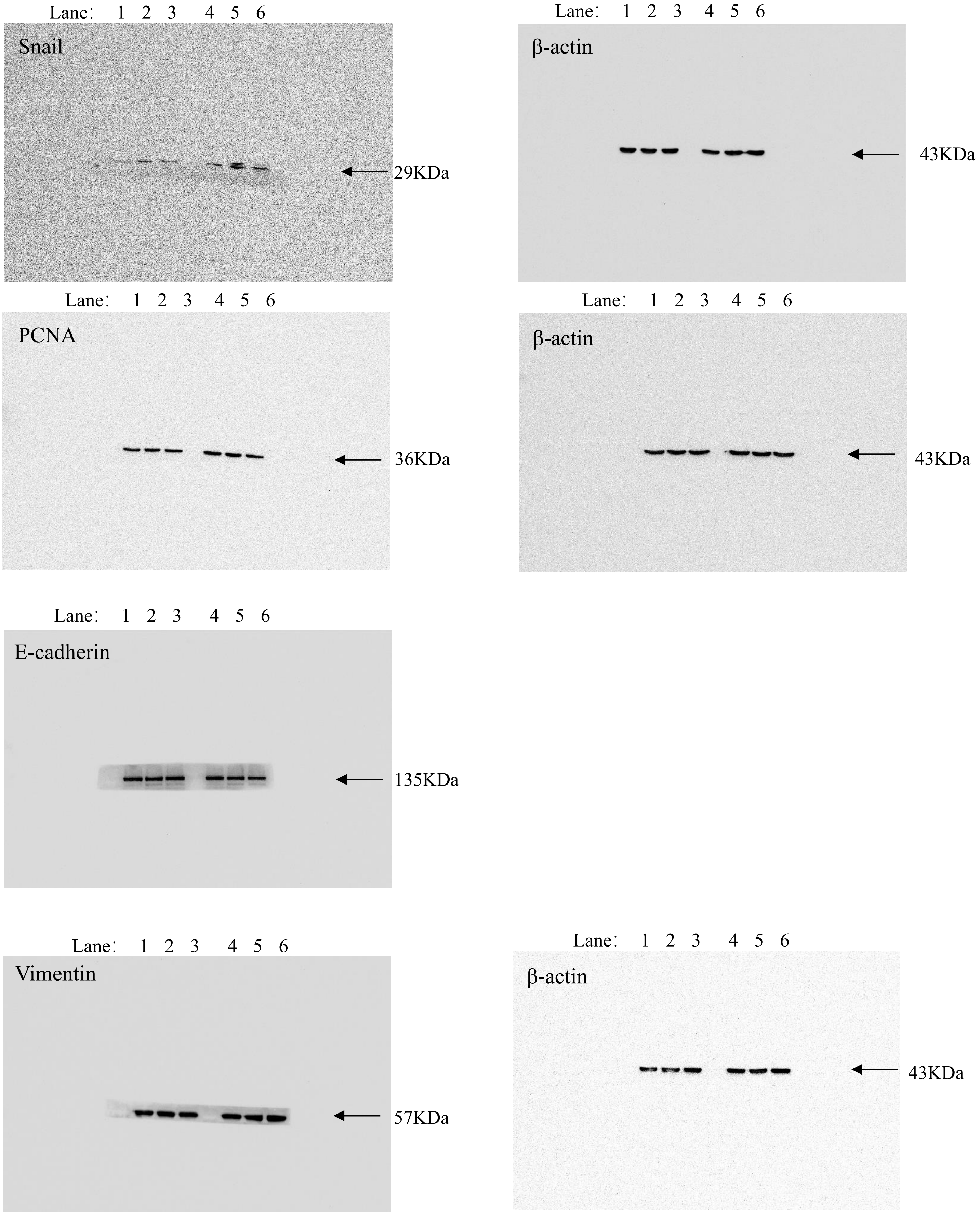

Lane: 1 NC 2: si-SorCS3-1# 3: si-SorCS3-2# 4: NC 5: si-SorCS3-1# 6: si-SorCS3-2#

Figure3-I

U251

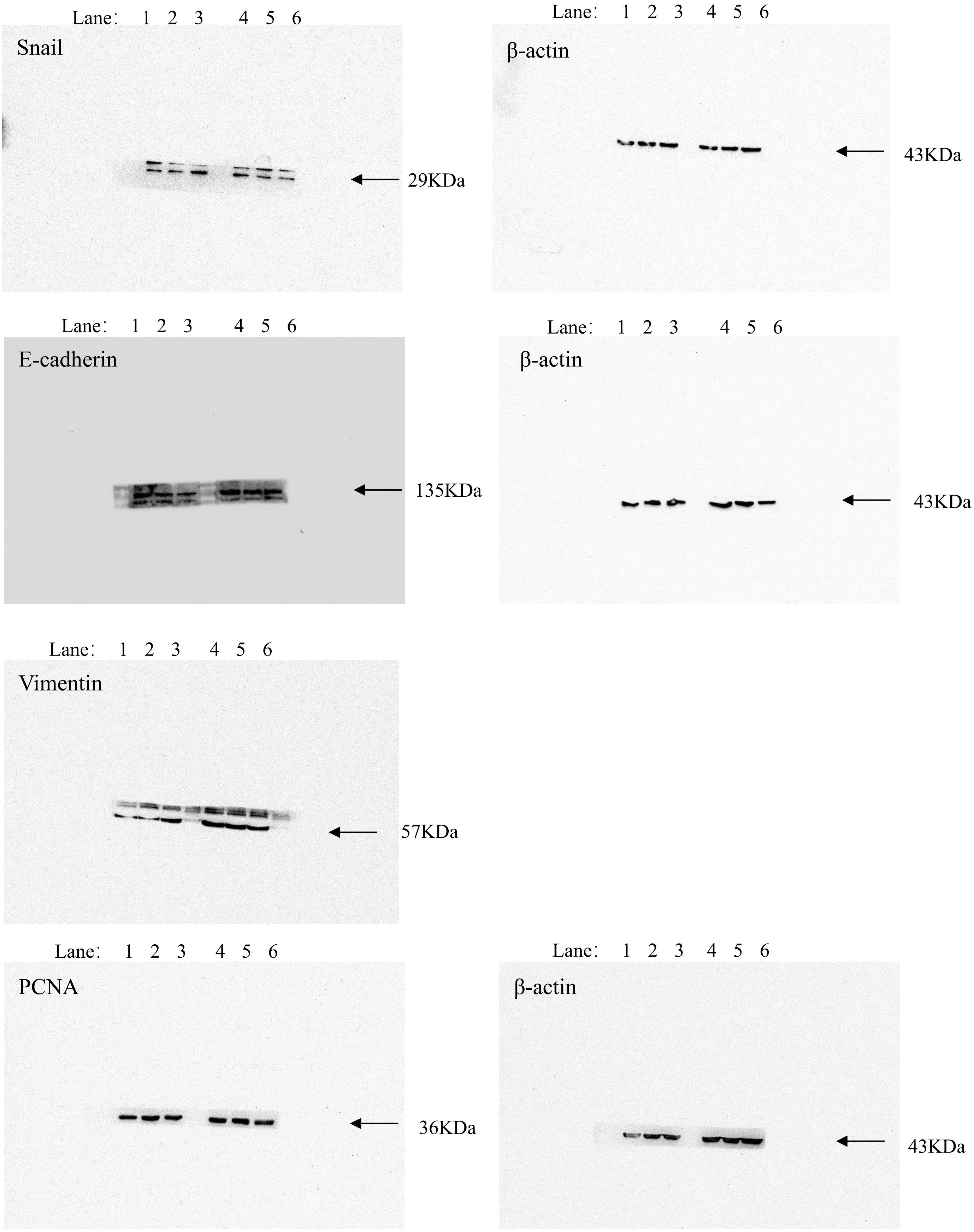

Lane: 1 NC 2: si-SorCS3-1# 3: si-SorCS3-2# 4: NC 5: si-SorCS3-1# 6: si-SorCS3-2#

Figure4-B

U87MG

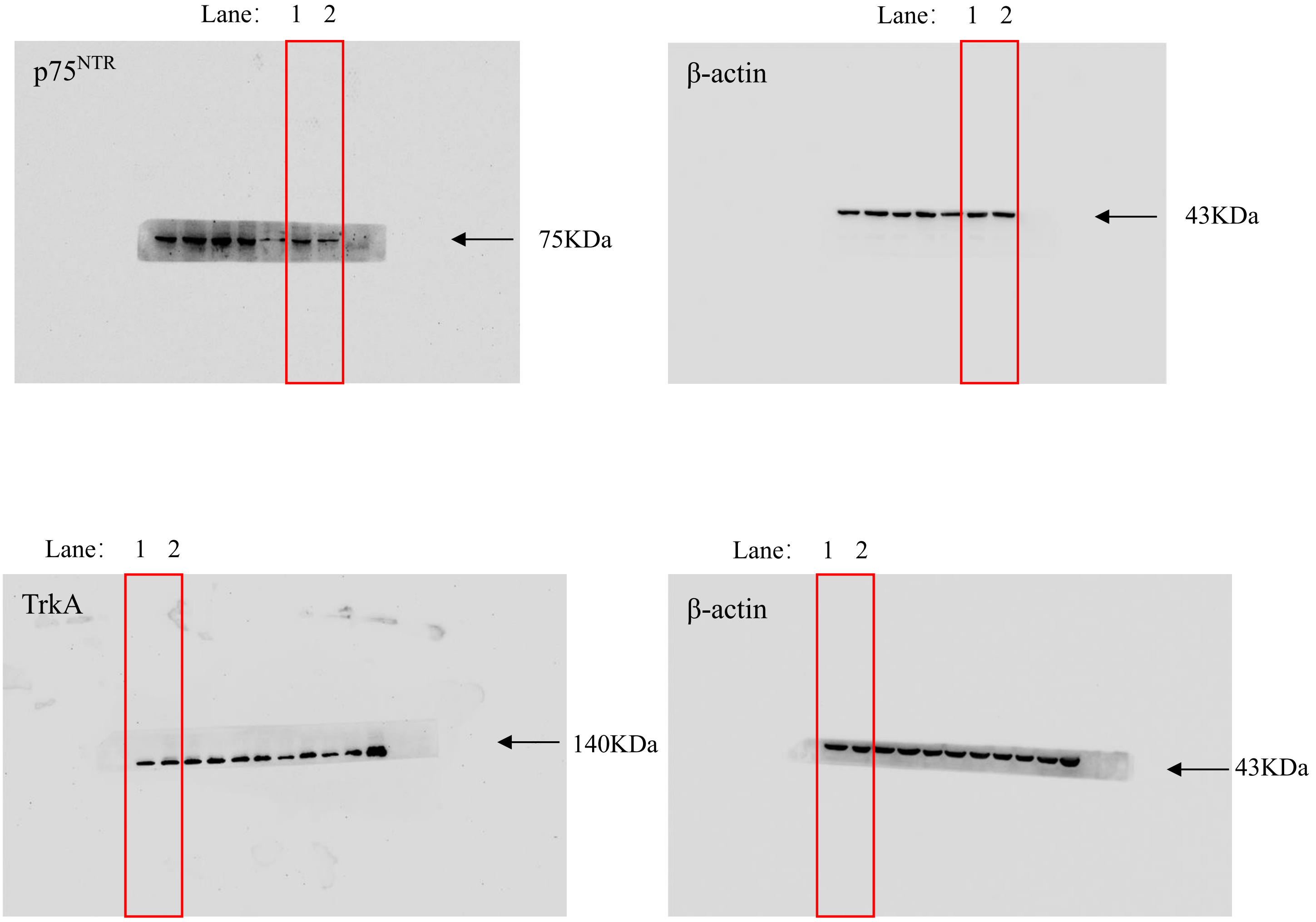

Lane: 1: NC 2: OE-SorSC3

Figure4-B

Lane: 1 2

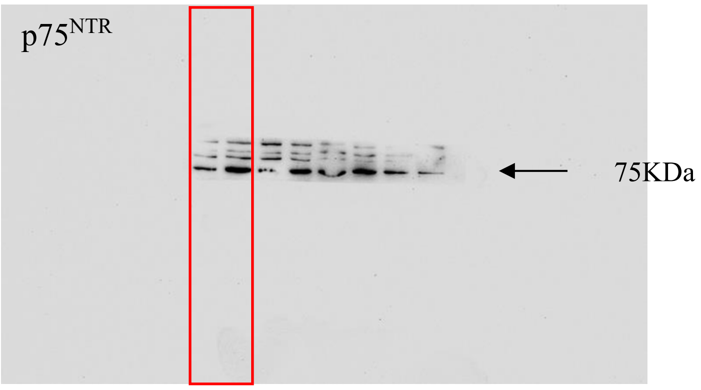

Lane: 1 2

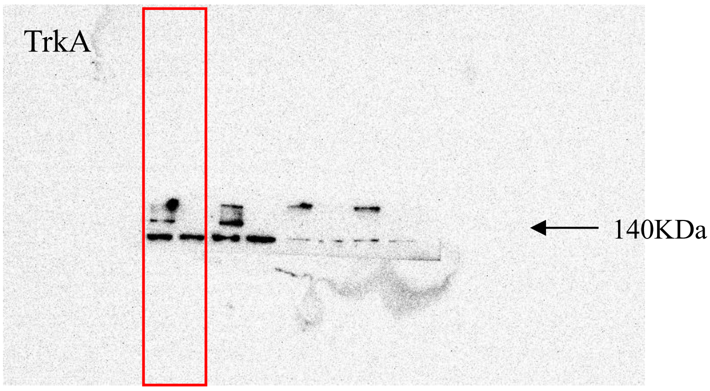

Lane: 1 2

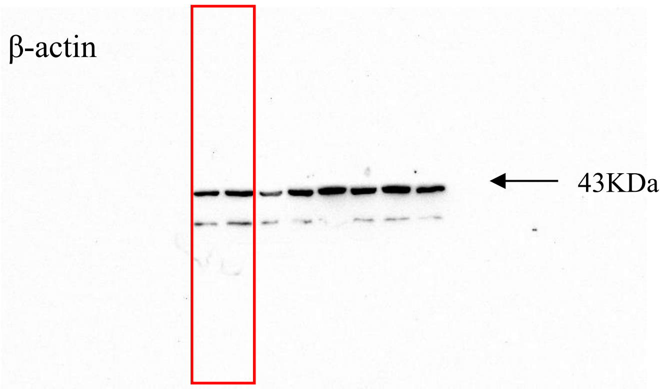

Lane: 1: NC 2: si-SorSC3-2#

Figure4-G Co-IP

Lane: 1 2 3 4 5 6

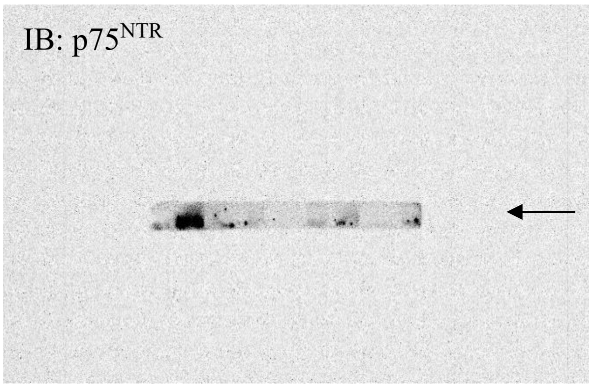

75KDa

IP Lane:
1: IgG
2: SorCS3-Flag
3: SorCS3-Flag
4: wash-1
5: wash-2
6: wash-3

Lane: 1 2 3 4 5 6

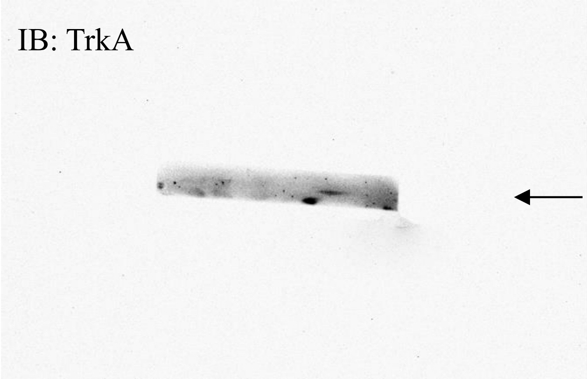

140KDa

Lane: 1: OE-SorSC3 2: NC 3: OE-SorCS3

Lane: 1 2 3 4 5 6 7 8

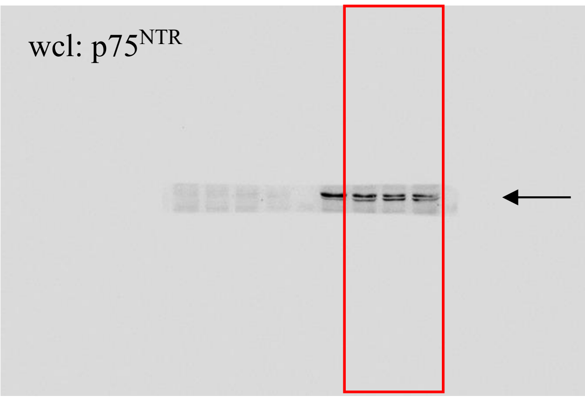

75KDa

Lane: 1 2 3 4 5 6 7 8

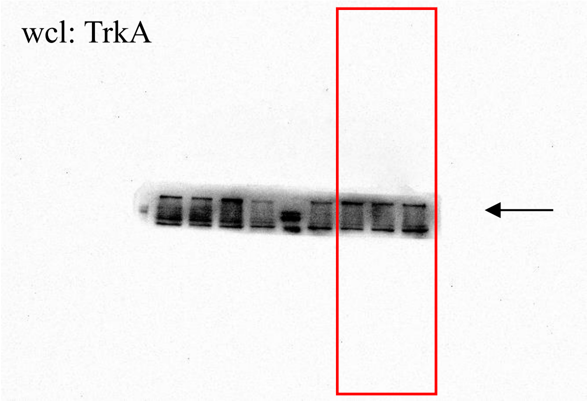

140KDa

Lane: 1 2 3 4 5 6 7 8

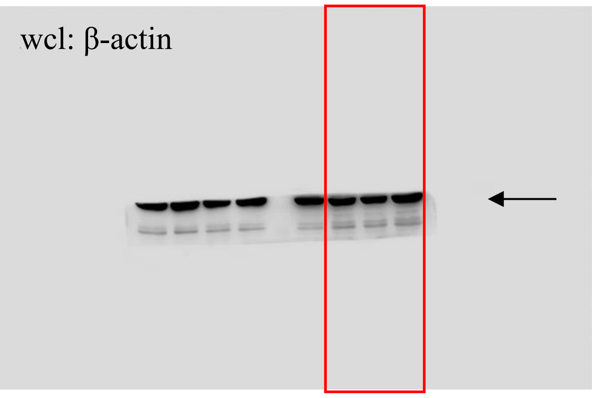

43KDa

Lane: 5: NC 6: OE-SorSC3 7: NC 8: OE-SorCS3

Figure4-H Co-IP

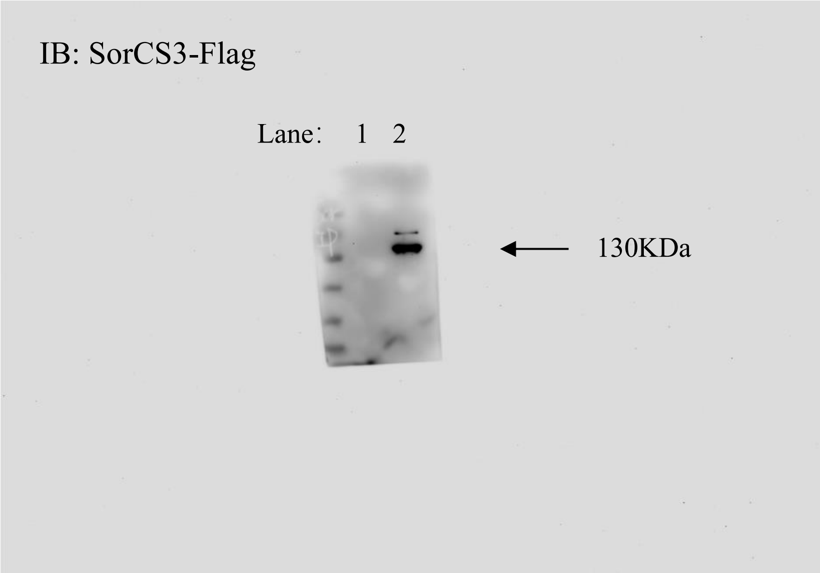

130KDa

IP Lane:
1: IgG
2: p75NTR

Lane: 1, 2: OE-SorCS3

Lane: 1 2

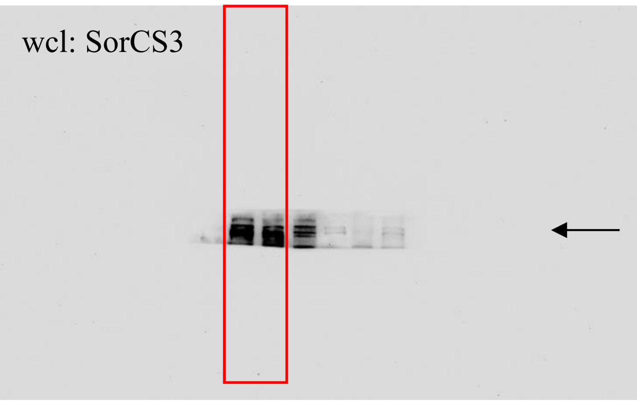

130KDa

Lane: 1 2

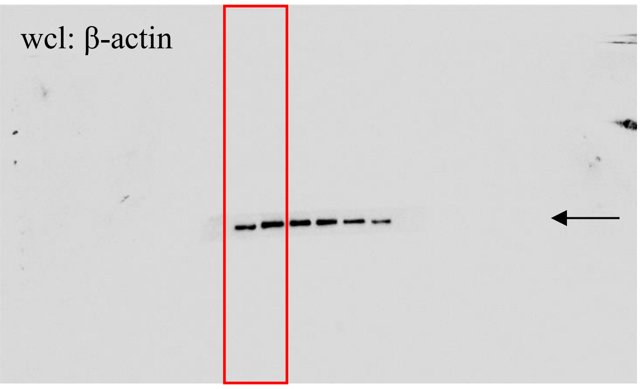

43KDa

Lane: 1, 2: OE-SorSC3

Figure5-A　Co-IP

Lane:　1　2　3　　4　5　6

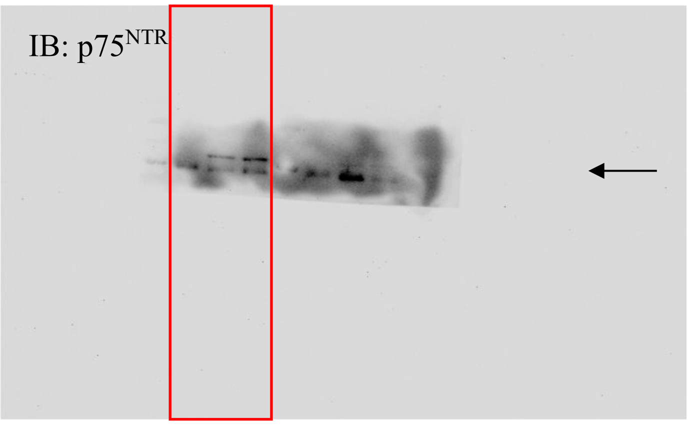

75KDa

IP　Lane:

1: IgG

2: SorCS3-Flag

3: SorCS3-Flag

4: IgG

5: SorCS3-Flag

6: SorCS3-Flag

Lane:　1: OE-SorSC3　2: OE-SorCS3　3: OE-SorCS3+NGF

Lane:　1　2　3　4　5　6

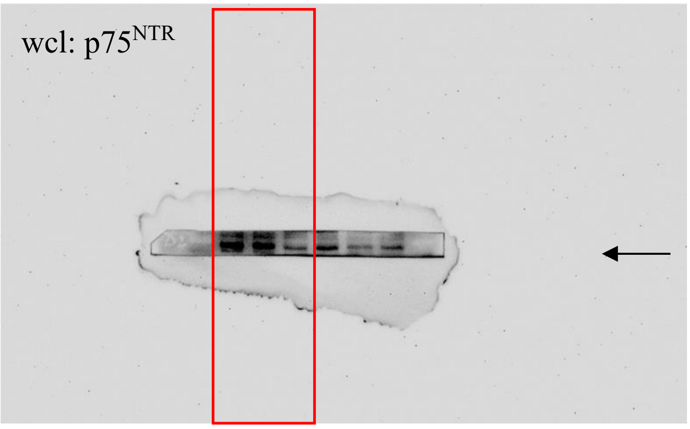

75KDa

Lane:　1　2　3　4　5　6

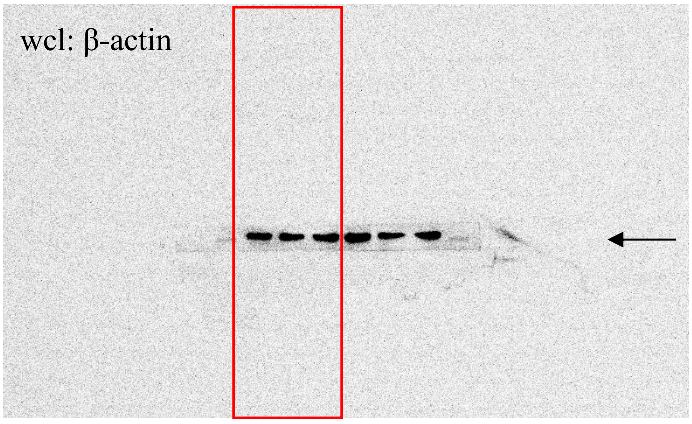

43KDa

Lane: 1: OE-SorSC3　2: OE-SorCS3　3: OE-SorCS3+NGF

Figure5-B

p75^{NTR}

Lane: 1 2 3 4 5 6 7 8 9 10 11 12

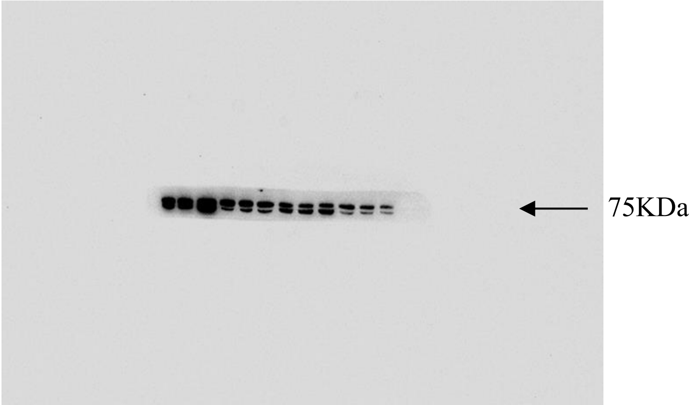

Lane: 1 2 3 4 5 6 7 8 9 10 11 12

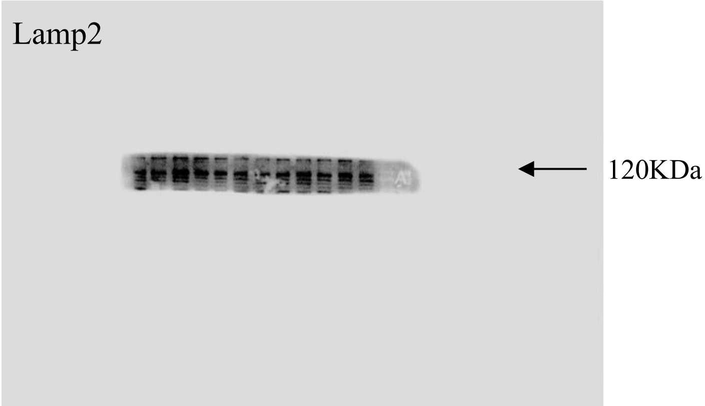

Lane: 1 2 3 4 5 6 7 8 9 10 11 12

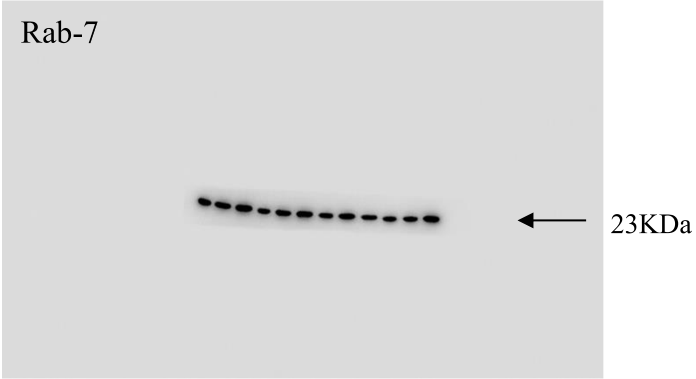

Lane: 1 2 3 4 5 6 7 8 9 10 11 12

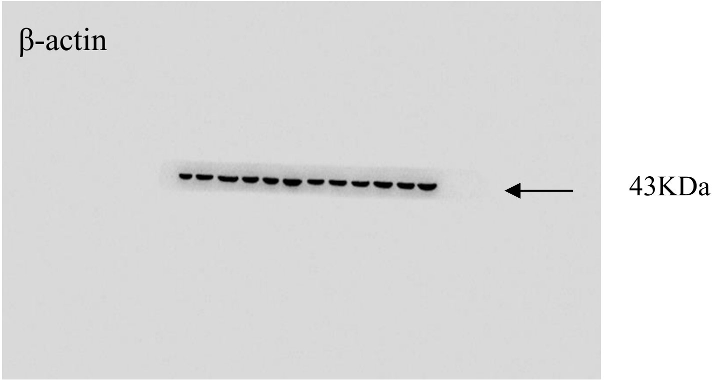

Lane:

1: NC+NGF(0 min)

2: NC+NGF(10 min)

3: NC+NGF(20 min)

4: NC+NGF(30 min)

5: NC+NGF(60 min)

6: NC+NGF(120 min)

7: OE-SorCS3+NGF(0 min)

8: OE-SorCS3+NGF(10 min)

9: OE-SorCS3+NGF(20 min)

10: OE-SorCS3+NGF(30 min)

11: OE-SorCS3+NGF(60 min)

12: OE-SorCS3+NGF(120 min)

Figure5-G OE-SorCS3

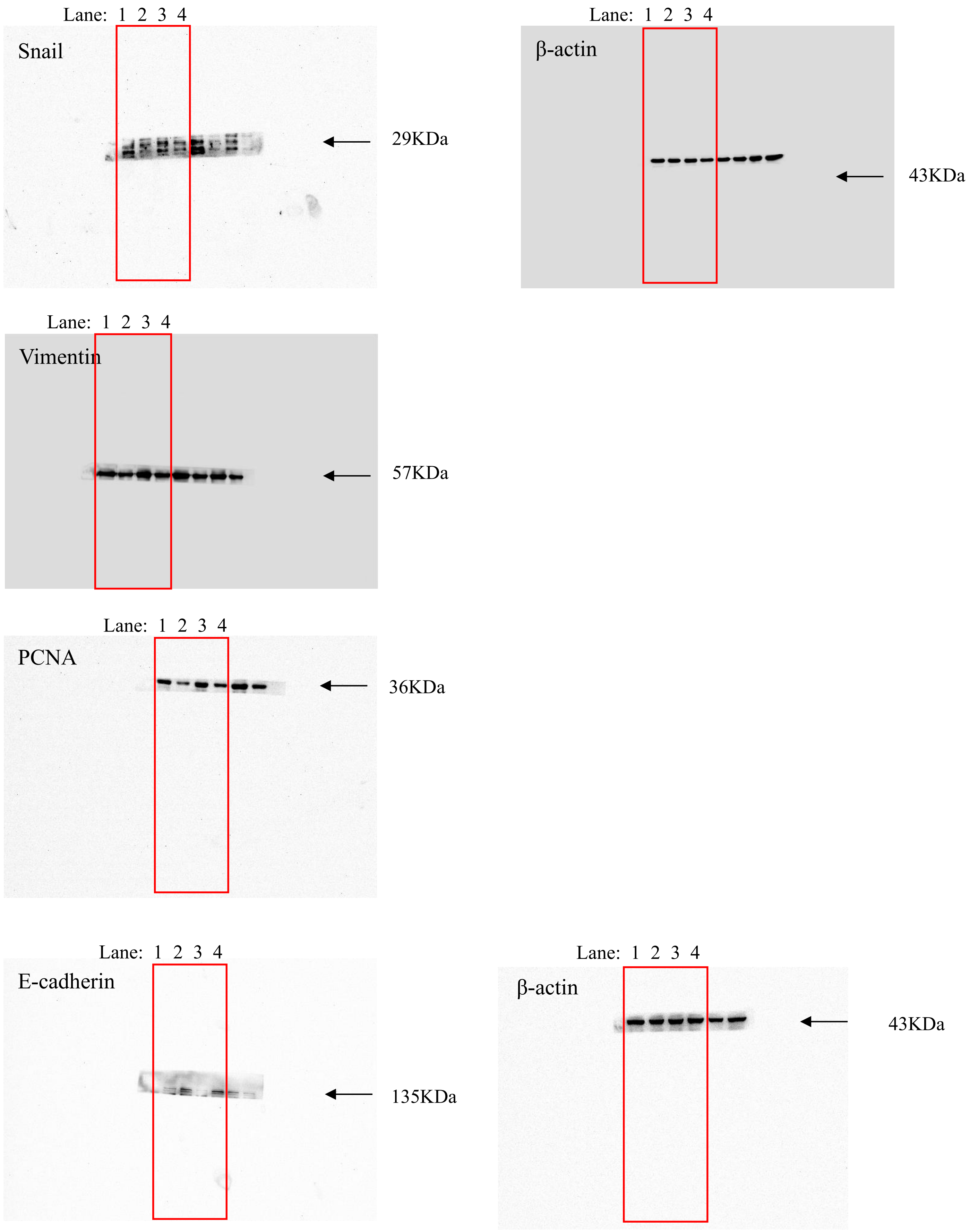

Lane: 1: NC 2: OE-SorCS3 3: NC+NGF 4: OE-SorCS3+NGF

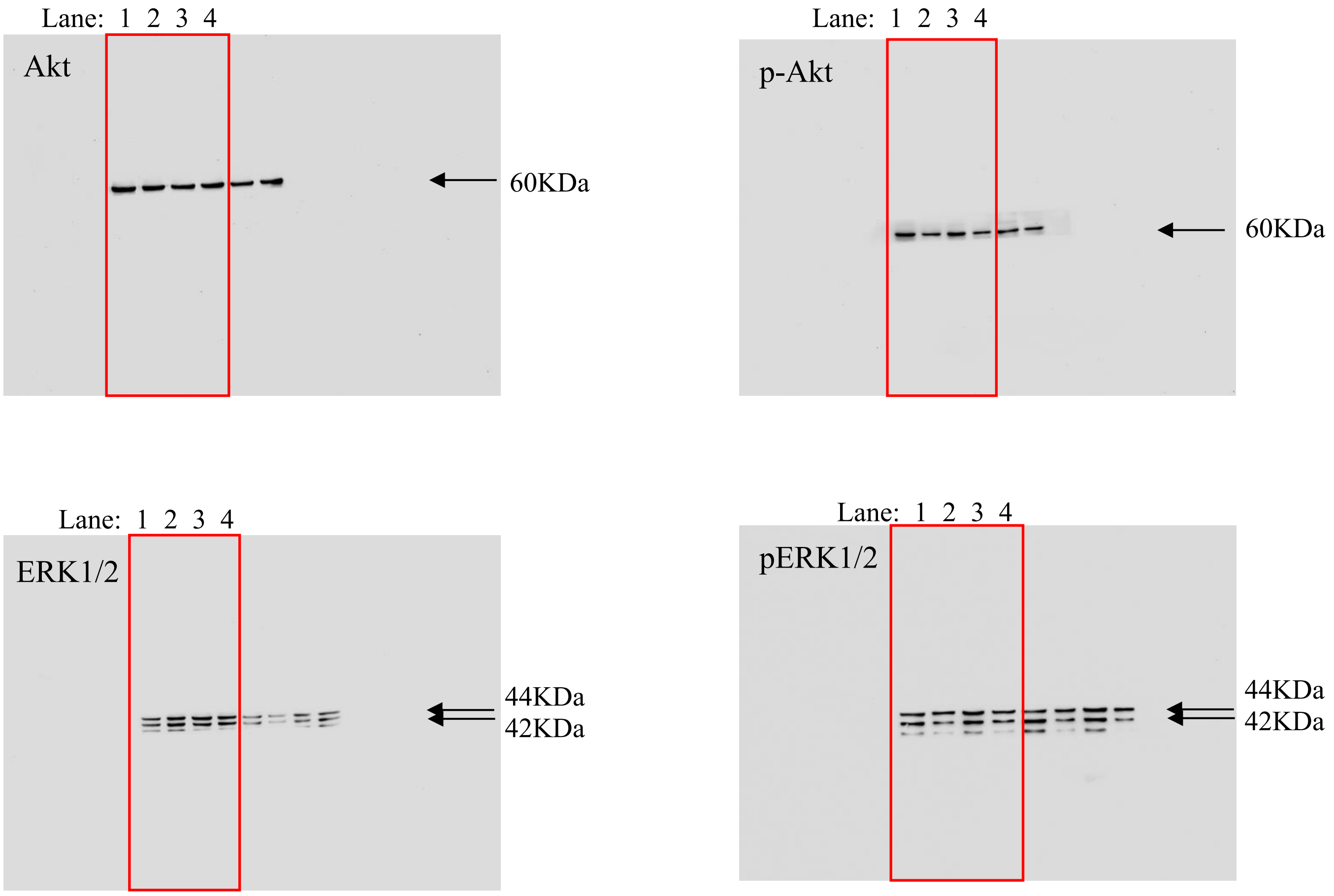

Lane: 1: NC 2: OE-SorCS3 3: NC+NGF 4: OE-SorCS3+NGF

Figure5-G si-SorCS3

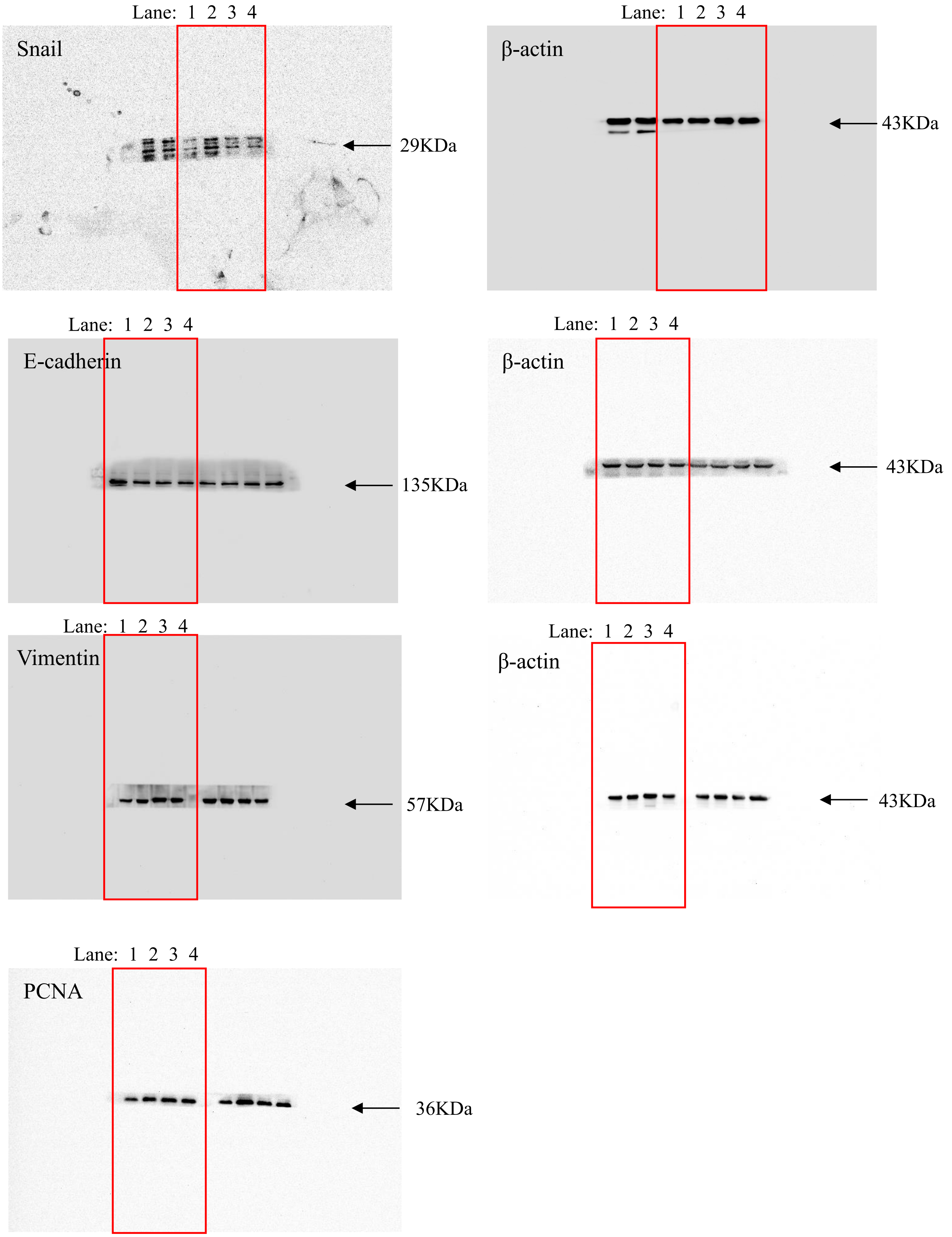

Lane: 1: NC 2: OE-SorCS3 3: NC+NGF 4: OE-SorCS3+NGF

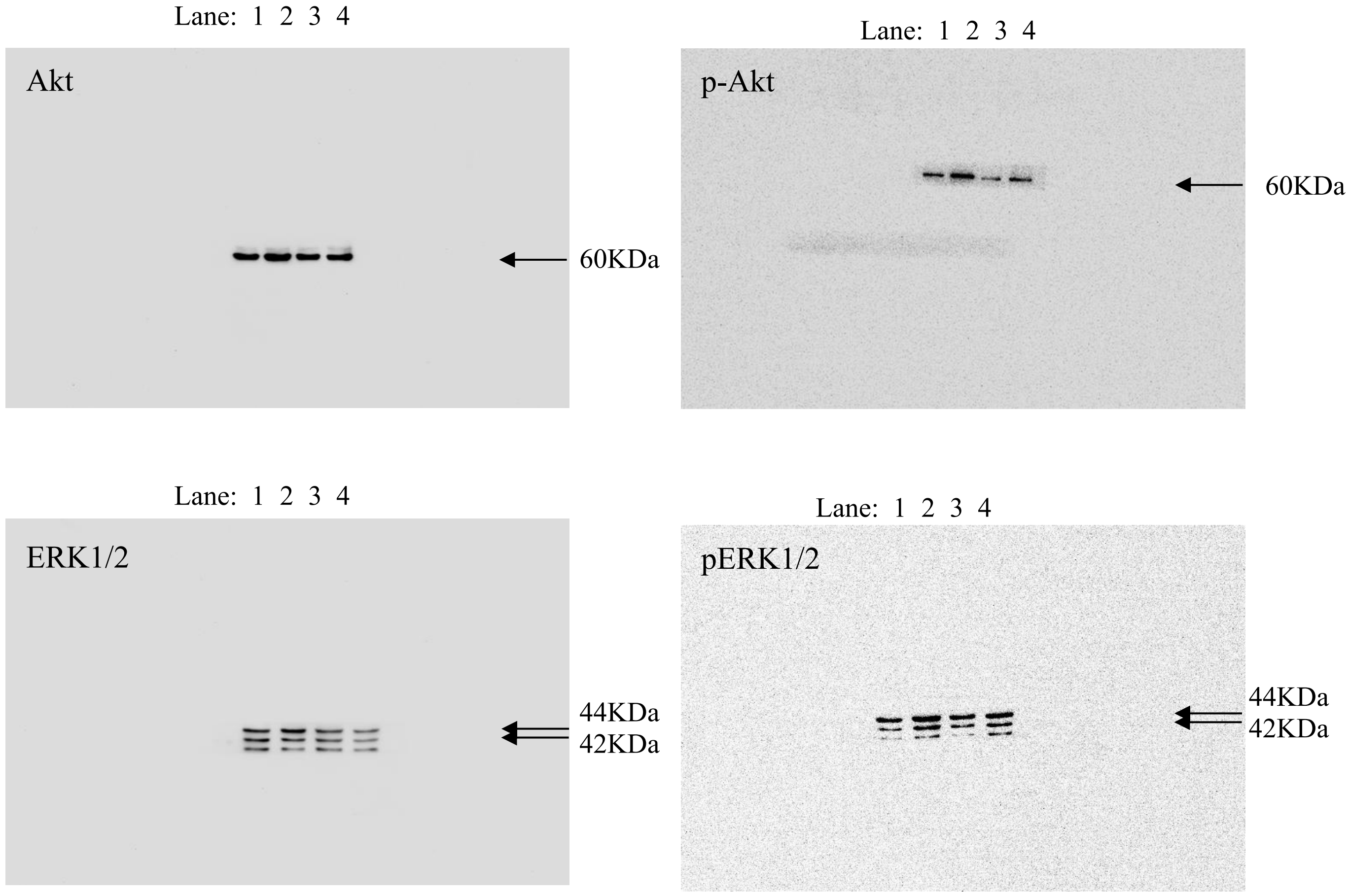

Lane: 1: NC   2: OE-SorCS3   3: NC+NGF   4: OE-SorCS3+NGF

Figure6-F

Lane: 1 2 3 4

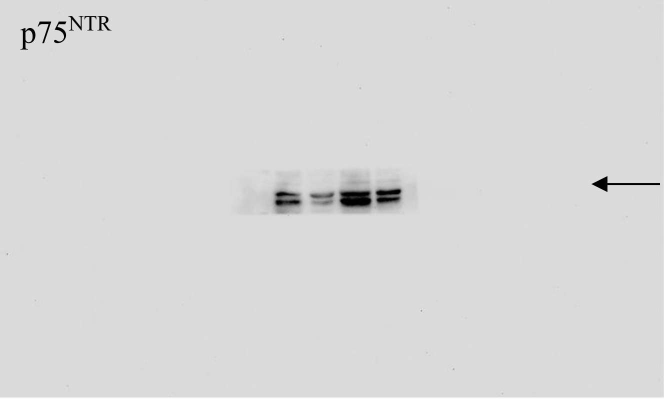

75KDa

Lane: 1 2 3 4

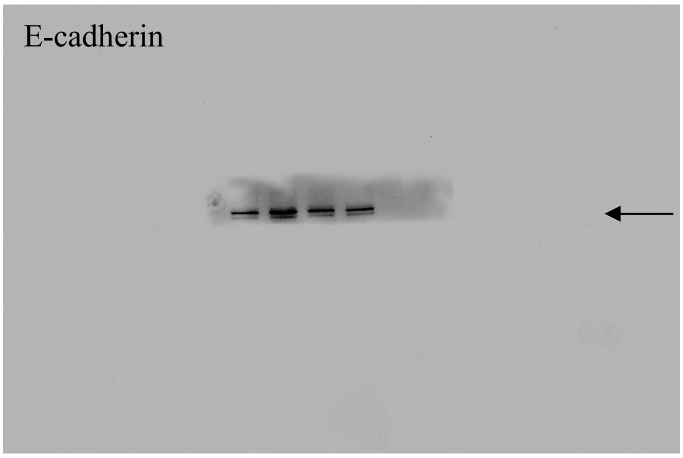

135KDa

Lane: 1 2 3 4

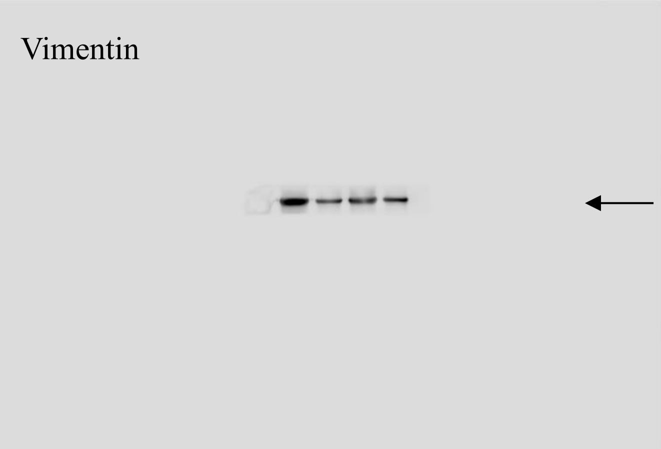

57KDa

Lane: 1 2 3 4

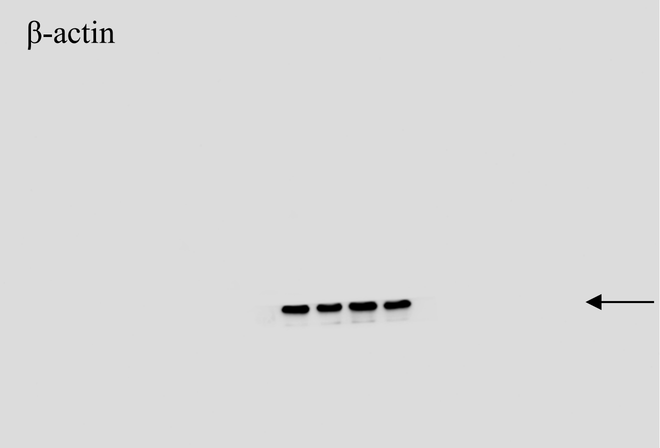

43KDa

Lane:

1: NC+NGF+DMSO

2: OE-SorSC3+NGF+DMSO

3: NC+NGF+Dynasore

4: OE-SorCS3+ NGF+Dynasore

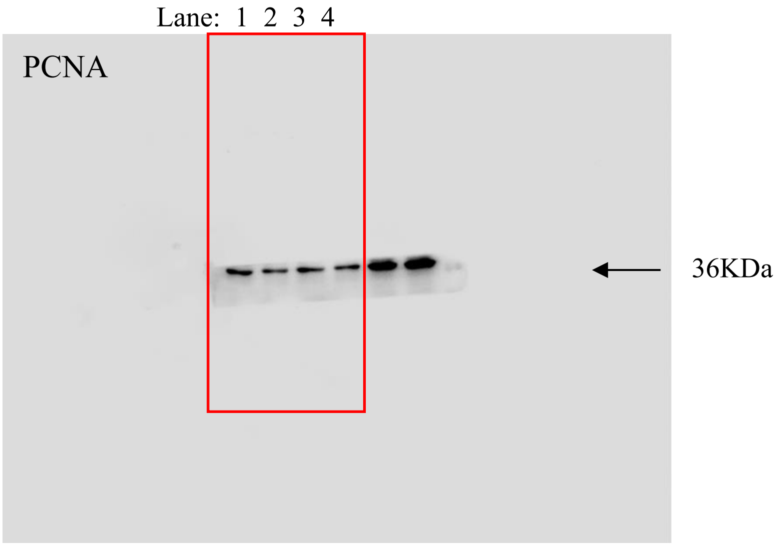

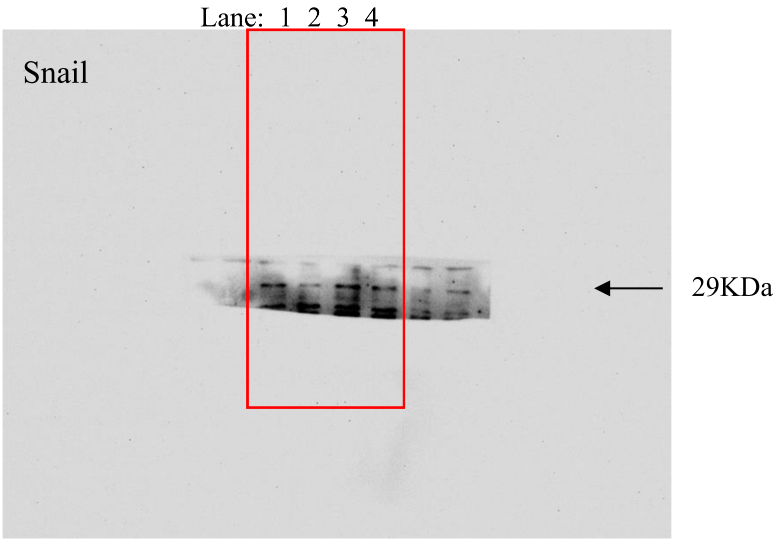

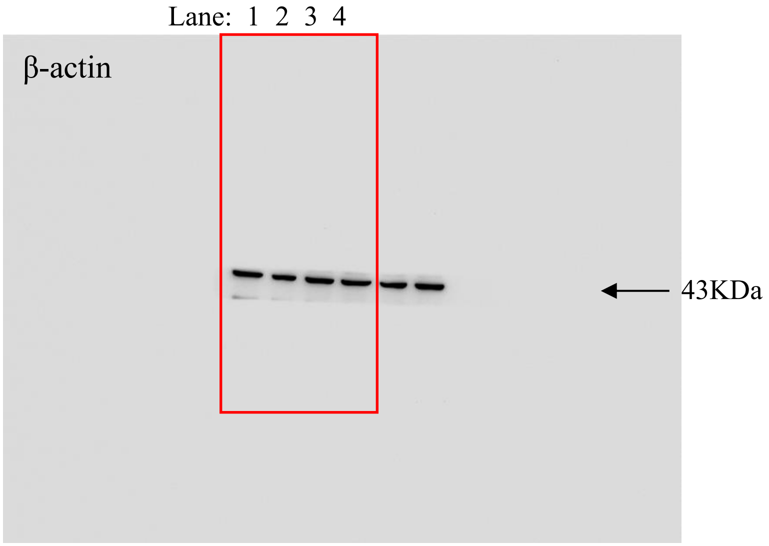

Lane: 1: NC+NGF+DMSO 2: OE-SorSC3+NGF+DMSO 3: NC+NGF+Dynasore 4: OE-SorCS3+ NGF+Dynasore

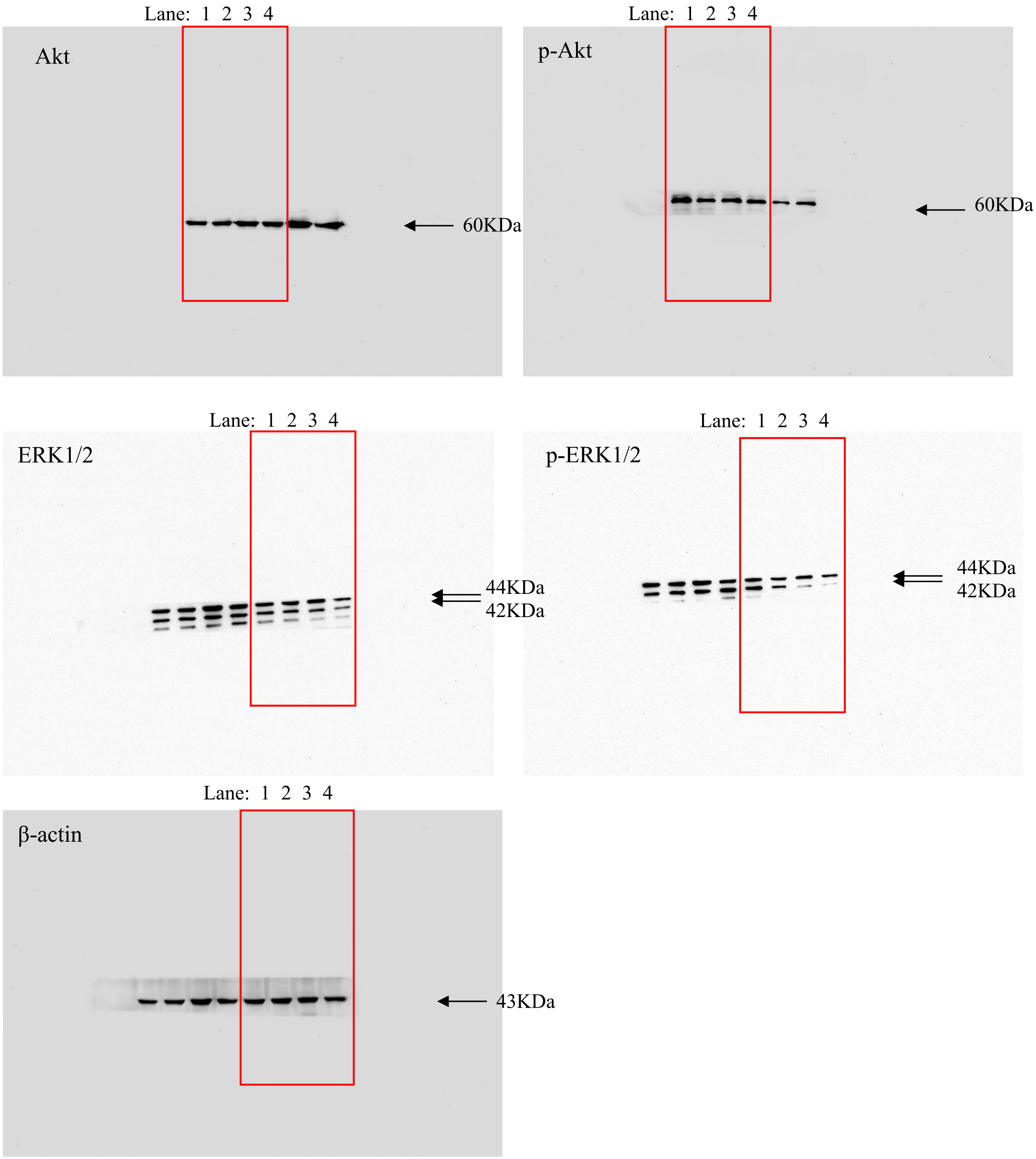

Lane: 1: NC+NGF+DMSO 2: OE-SorSC3+NGF+DMSO 3: NC+NGF+Dynasore 4: OE-SorCS3+ NGF+Dynasore

Figure6-I

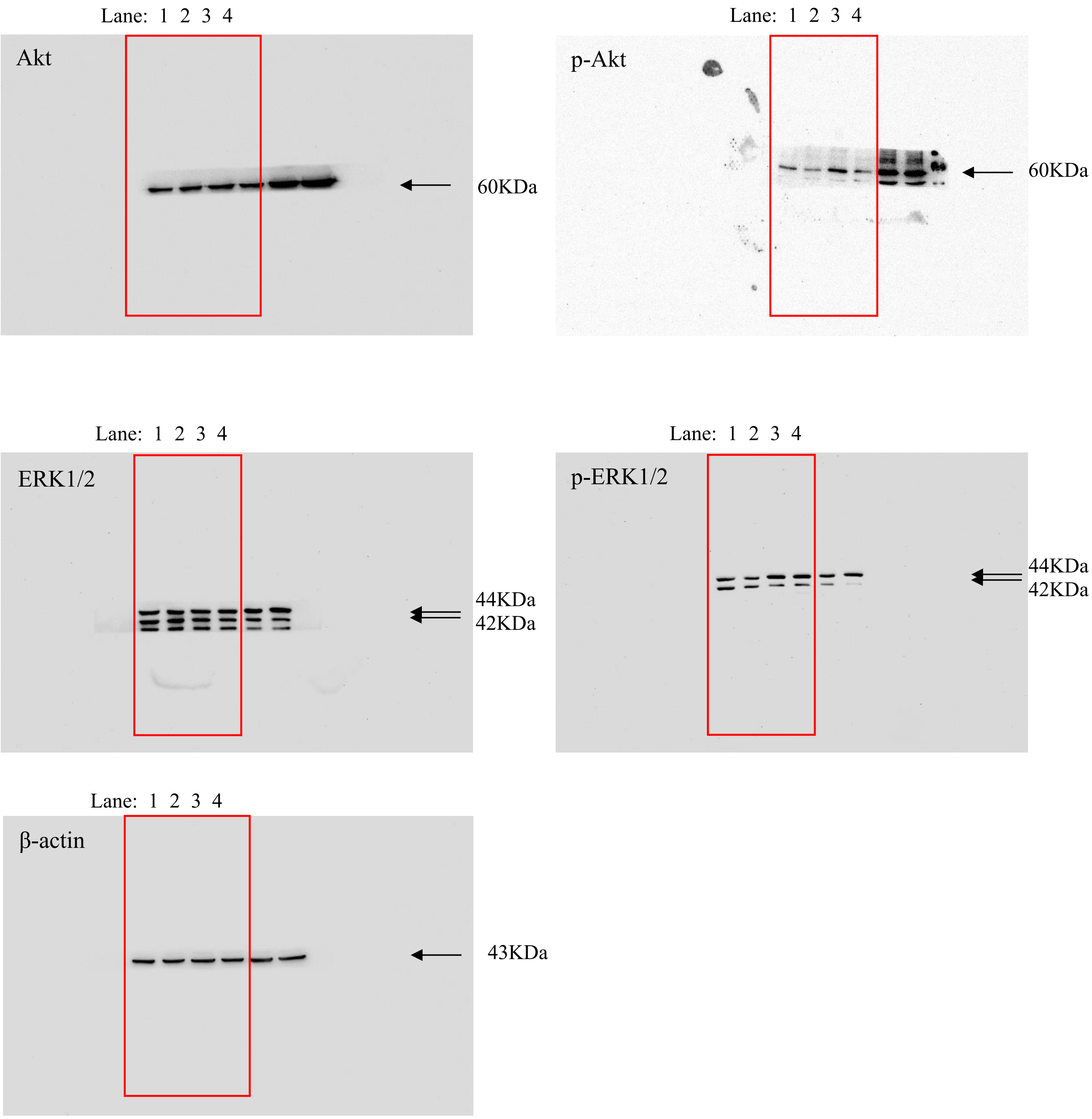

Lane: 1: NC 2: OE-SorSC3 3: NC+Ro 08-2750 4: OE-SorCS3+Ro 08-2750

Supplementary Figure1-A

Lane: 1 2 3 4

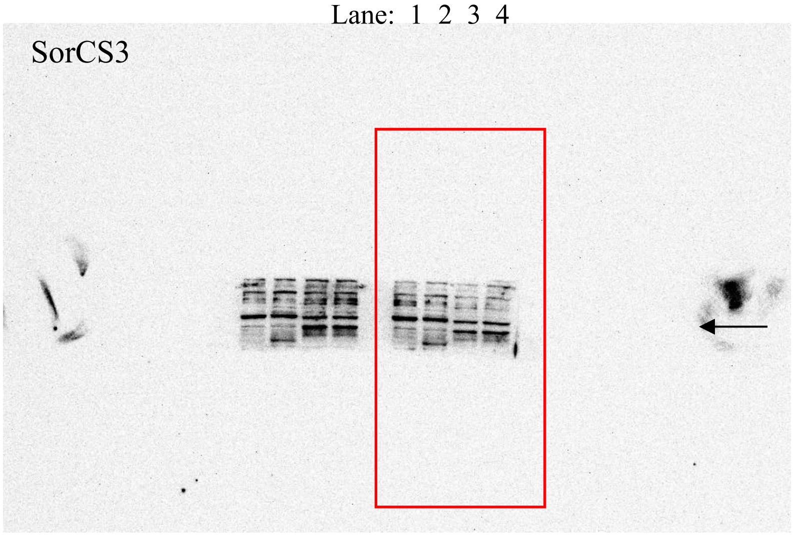

130KDa

Lane: 1 2 3 4

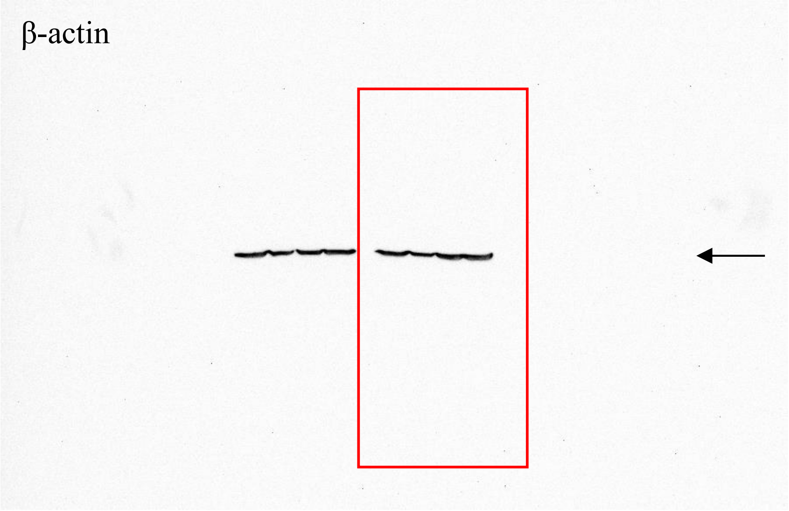

43KDa

Lane: 1: U251 2: A172 3: T98 4: U87MG

Supplementary Figure1-B

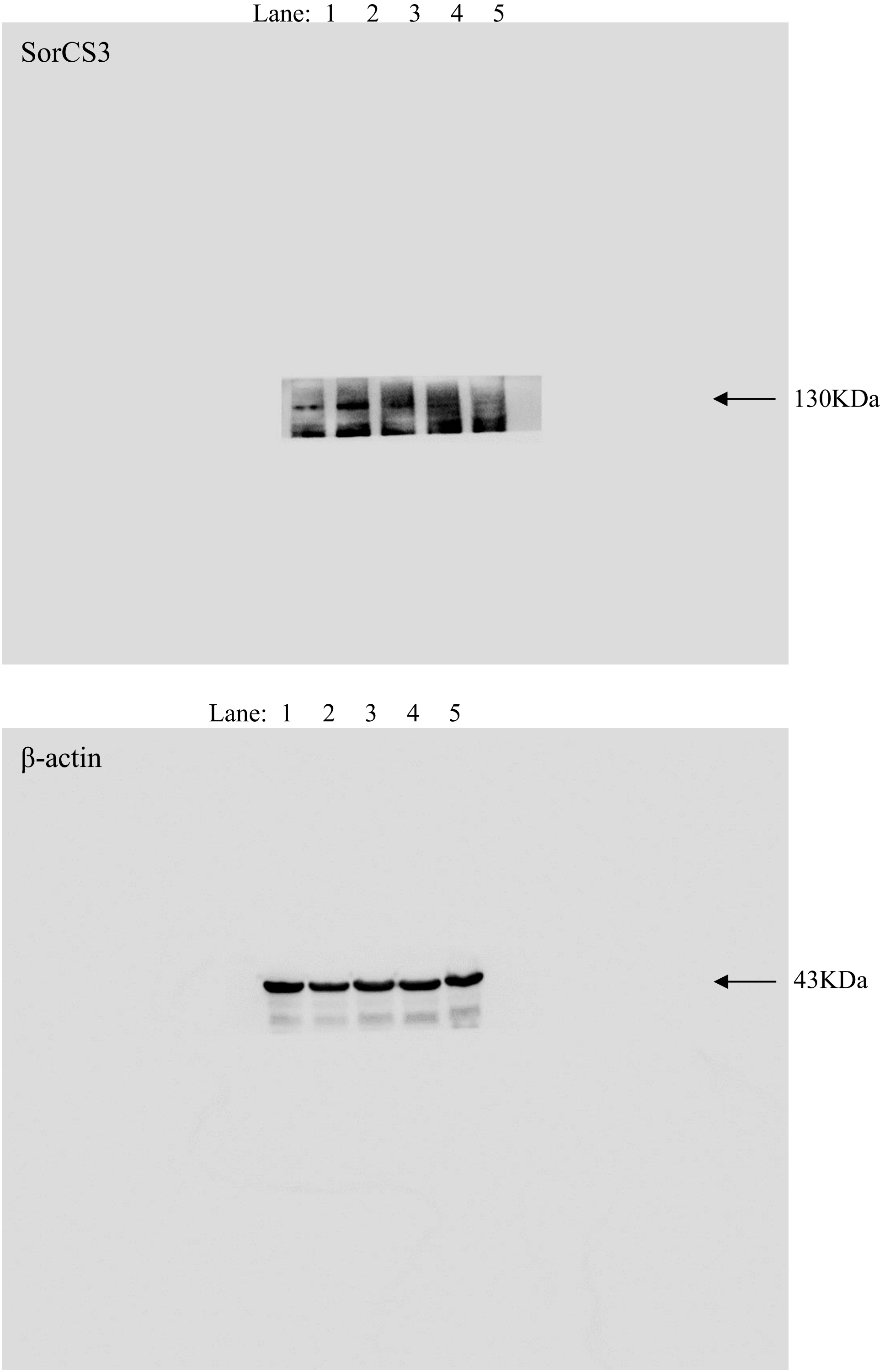

Lane: 1: NC 2: OE-SorCS3 3: NC 4: si-SorCS3-1# 5: siSorCS3-2#

Supplementary Figure2-A

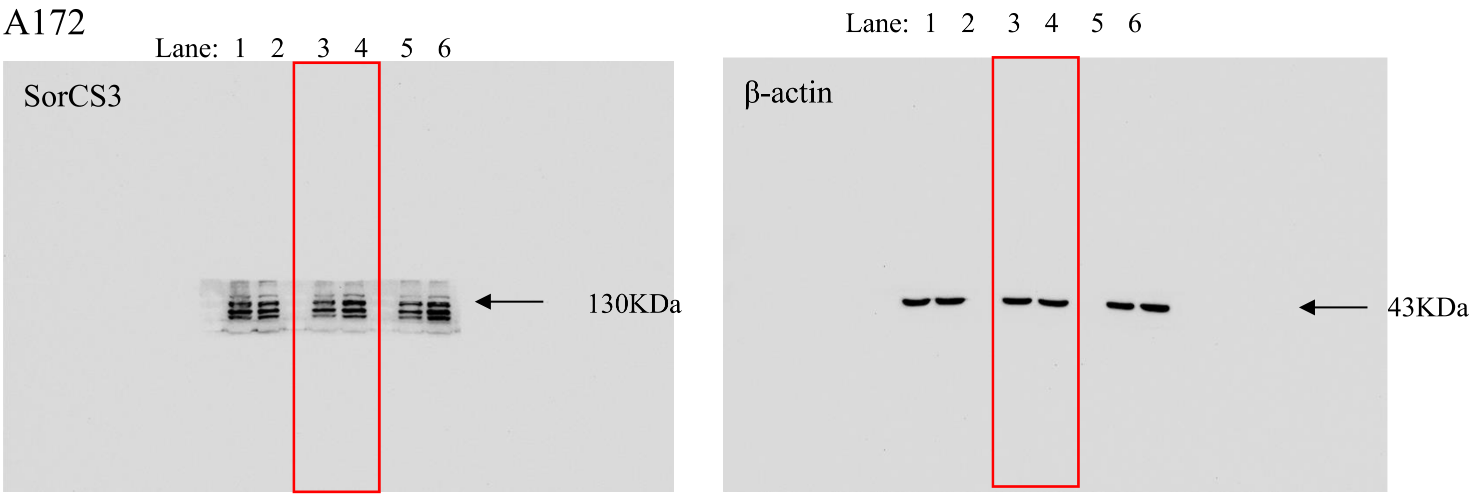

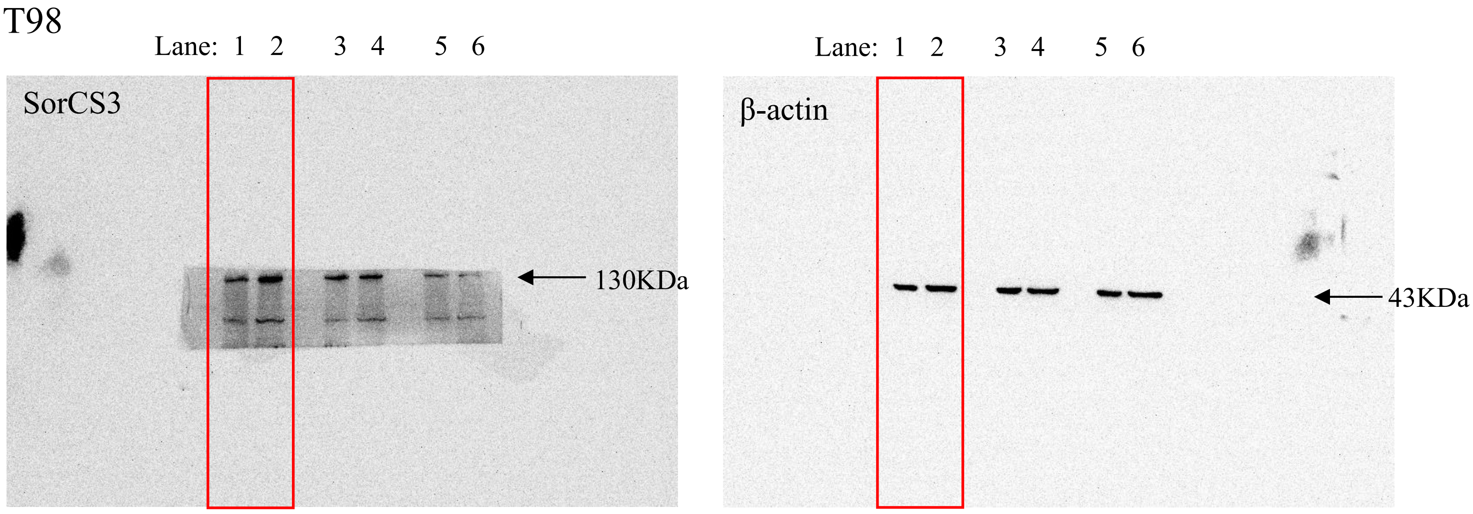

Lane: 1, 3, 5: NC 2, 4, 6: OE-SorSC3-Flag
